# A method to remove the influence of fixative concentration on postmortem T_2_ maps using a kinetic tensor model

**DOI:** 10.1002/hbm.25661

**Published:** 2021-09-20

**Authors:** Benjamin C. Tendler, Feng Qi, Sean Foxley, Menuka Pallebage‐Gamarallage, Ricarda A. L. Menke, Olaf Ansorge, Samuel A. Hurley, Karla L. Miller

**Affiliations:** ^1^ Wellcome Centre for Integrative Neuroimaging, FMRIB, Nuffield Department of Clinical Neurosciences University of Oxford Oxford; ^2^ Department of Radiology University of Chicago Chicago Illinois USA; ^3^ Nuffield Department of Clinical Neurosciences University of Oxford Oxford UK; ^4^ Department of Radiology University of Wisconsin–Madison Madison Wisconsin USA

**Keywords:** 7 T, diffusion MRI, fixative modelling, formalin, histology, post‐mortem human brain, T_2_ mapping

## Abstract

Formalin fixation has been shown to substantially reduce T_2_ estimates, primarily driven by the presence of fixative in tissue. Prior to scanning, post‐mortem samples are often placed into a fluid that has more favourable imaging properties. This study investigates whether there is evidence for a change in T_2_ in regions close to the tissue surface due to fixative outflux into this surrounding fluid. Furthermore, we investigate whether a simulated spatial map of fixative concentration can be used as a confound regressor to reduce T_2_ inhomogeneity. To achieve this, T_2_ maps and diffusion tensor estimates were obtained in 14 whole, formalin‐fixed post‐mortem brains placed in Fluorinert approximately 48 hr prior to scanning. Seven brains were fixed with 10% formalin and seven brains were fixed with 10% neutral buffered formalin (NBF). Fixative outflux was modelled using a proposed kinetic tensor (KT) model, which incorporates voxelwise diffusion tensor estimates to account for diffusion anisotropy and tissue‐specific diffusion coefficients. Brains fixed with 10% NBF revealed a spatial T_2_ pattern consistent with modelled fixative outflux. Confound regression of fixative concentration reduced T_2_ inhomogeneity across both white and grey matter, with the greatest reduction attributed to the KT model versus simpler models of fixative outflux. No such effect was observed in brains fixed with 10% formalin. Correlations between the transverse relaxation rate *R*_2_ and ferritin/myelin proteolipid protein (PLP) histology lead to an increased similarity for the relationship between *R*_2_ and PLP for the two fixative types after KT correction.

## Graphical abstract

Fixed post‐mortem tissue samples are often placed into a fluid with more favourable imaging properties prior to MRI scanning. This study investigates whether there is evidence for a change in T_2_ in regions close to the tissue surface due to fixative outflux into this surrounding fluid, and whether a simulated spatial map of fixative concentration can be used as a confound regressor to reduce T_2_ inhomogeneity. Modelling fixative outflux using a proposed kinetic tensor (KT) model, we identified a spatial T_2_ pattern consistent with modelled fixative outflux, and demonstrated that regressing fixative concentration reduced T_2_ inhomogeneity across both white and grey matter in brains fixed with 10% neutral buffered formalin (NBF).

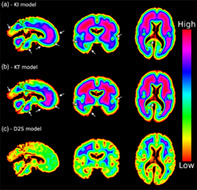

## 1 INTRODUCTION

Post‐mortem imaging allows for the acquisition of high‐resolution datasets and validation of the origin of image contrast through comparisons with histology. However, fresh tissue samples are vulnerable to damage through mechanical handling and decomposition though autolysis and putrefaction (Thavarajah, Mudimbaimannar, Elizabeth, Rao, & Ranganathan, 2012). To prevent this, samples are often first fixed prior to imaging using an aldehyde (Kiernan, 2000) solution such as formalin (Fox, Johnson, Whiting, & Roller, 1985), to prevent decomposition and improve mechanical strength and stability.

Fixation has been shown to have a substantial effect on MR‐relevant tissue properties, with decreases in T_1_ (Birkl et al., 2016, 2018; Kamman, Go, Stomp, Hulstaert, & Berendsen, 1985; Nagara et al., 1987; Pfefferbaum, Sullivan, Adalsteinsson, Garrick, & Harper, 2004; Shepherd, Thelwall, et al., 2009a), T_2_ (Birkl et al., 2016; Birkl et al., 2018; Dawe, Bennett, Schneider, Vasireddi, & Arfanakis, 2009; Kamman et al., 1985; Nagara et al., 1987; Pfefferbaum et al., 2004; Shepherd, Thelwall, et al., 2009a; Thelwall, Shepherd, Stanisz, & Blackband, 2006), T_2_* (Birkl et al., 2016, 2018) and diffusivity (D'Arceuil, Westmoreland, & de Crespigny, 2007; Shepherd, Thelwall, et al., 2009a; Sun et al., 2005; Sun, Neil, & Song, 2003; Thelwall et al., 2006) reported. These changes are thought to arise through either reactions with the aldehyde fixative solution in tissue via protein cross‐linking (Kiernan, 2000) or presence of the fixative solution (Shepherd, Thelwall, et al., 2009a; fixative that has been absorbed into tissue). These changes have been shown to depend on fixative type, concentration and vendor‐specific composition (Birkl et al., 2018; Shepherd, Thelwall, et al., 2009a; Thelwall et al., 2006).

To improve the signal‐to‐noise ratio (SNR), post‐mortem samples are often first “washed” via immersion in an external medium such as phosphate buffered saline (PBS), leading to exchange between the external medium and the fixative solution. This process has been shown to restore T_2_ values close to those obtained prior to fixation (Shepherd, Thelwall, et al., 2009a), indicating that the change in T_2_ (due to fixation) is primarily driven by the presence of fixative within tissue, rather than changes to the tissue itself. For formalin specifically, the decrease in T_2_ has been estimated as linearly dependent on its concentration (Shepherd, Thelwall, et al., 2009a).

In addition to washing the post‐mortem tissue samples, it has become increasingly commonplace to place tissue samples in an alternative fluid during scanning that has more favourable properties for imaging (Dusek et al., 2019). One example is fluorinert (3M), a susceptibility‐matched perfluorocarbon fluid that produces no signal in MR images. This makes it possible to perform scanning without having to adapt protocols in light of signal from the surrounding medium (e.g., it is possible to perform imaging experiments considering a field‐of‐view that only covers the tissue sample) and obtain images that have minimal susceptibility‐induced distortions or other artefacts.

Large samples (such as whole human post‐mortem brains) are often not washed (Miller et al., 2011), due to the prohibitive length of time required for the external medium to penetrate into deep tissue. Assuming timescales for PBS are similar, it is informative that formalin takes weeks to fully penetrate human brains during immersion fixation (Dawe et al., 2009; Yong‐Hing, Obenaus, Stryker, Tong, & Sarty, 2005). This can result in unwanted hydration boundaries that alter MR contrast (Miller et al., 2011) and would compromise quantitative MRI estimates.

Large tissue samples can still be placed within an alternative fluid prior to scanning to improve the imaging environment (Dusek et al., 2019). When considering formalin‐fixed tissue, if there is any outflux of formalin into this surrounding medium, this may lead to a reduced concentration and a change in T_2_ in regions with close proximity to the brain surface. While this would be expected to be less problematic than the hydration boundaries mentioned above, it could still have a confounding effect on quantitative estimates. In this study, we investigate whether there is evidence for such an effect in T_2_ maps acquired in whole, formalin‐fixed, human post‐mortem brains placed in fluorinert approximately 48 hr prior to scanning. We simulate the outflux of fixative at the tissue surface and compare the resulting concentration distribution to the T_2_ values across our brain. We simulate outflux using a model that incorporates the effects of diffusion anisotropy and tissue specific diffusion coefficients, which aims to provide realistic modelling of fixative dynamics (fixative flux) within different tissue types.

Previous studies of fixative dynamics (Dawe et al., 2009; Yong‐Hing, Obenaus, Stryker, Tong, & Sarty, 2005) have aimed to characterise how the process of fixation and presence of fixative influences MRI parameters. Here, we take this approach one step further and propose that the resulting map of fixative concentration can be used as a confound regressor to account (or correct) for the effects of fixative concentration on T_2_. The correction is performed using a single global regressor that is fit to the T_2_ map across all of white matter. The importance of such a correction is evaluated by comparing the homogeneity of T_2_ estimates over white and grey matter separately within the post‐mortem brains before and after correction. Furthermore, the importance of incorporating a more realistic model of fixative dynamics (including the effects of both diffusion anisotropy and tissue‐specific diffusion coefficients) is compared against two alternative models, the first incorporating isotropic diffusion and a uniform diffusion coefficient, and the second based on a distance‐to‐surface model. We evaluate this correction in a cohort of brains fixed with two types of fixative, 10% formalin and 10% neutral buffered formalin (NBF). Finally, resulting transverse relaxation rate (*R*_2_) estimates before and after correction are correlated with histological measurements of ferritin and myelin proteolipid protein (PLP) content obtained within the same brain.

## 2 THEORY

### 2.1 The kinetic tensor model

**FIGURE 1:**
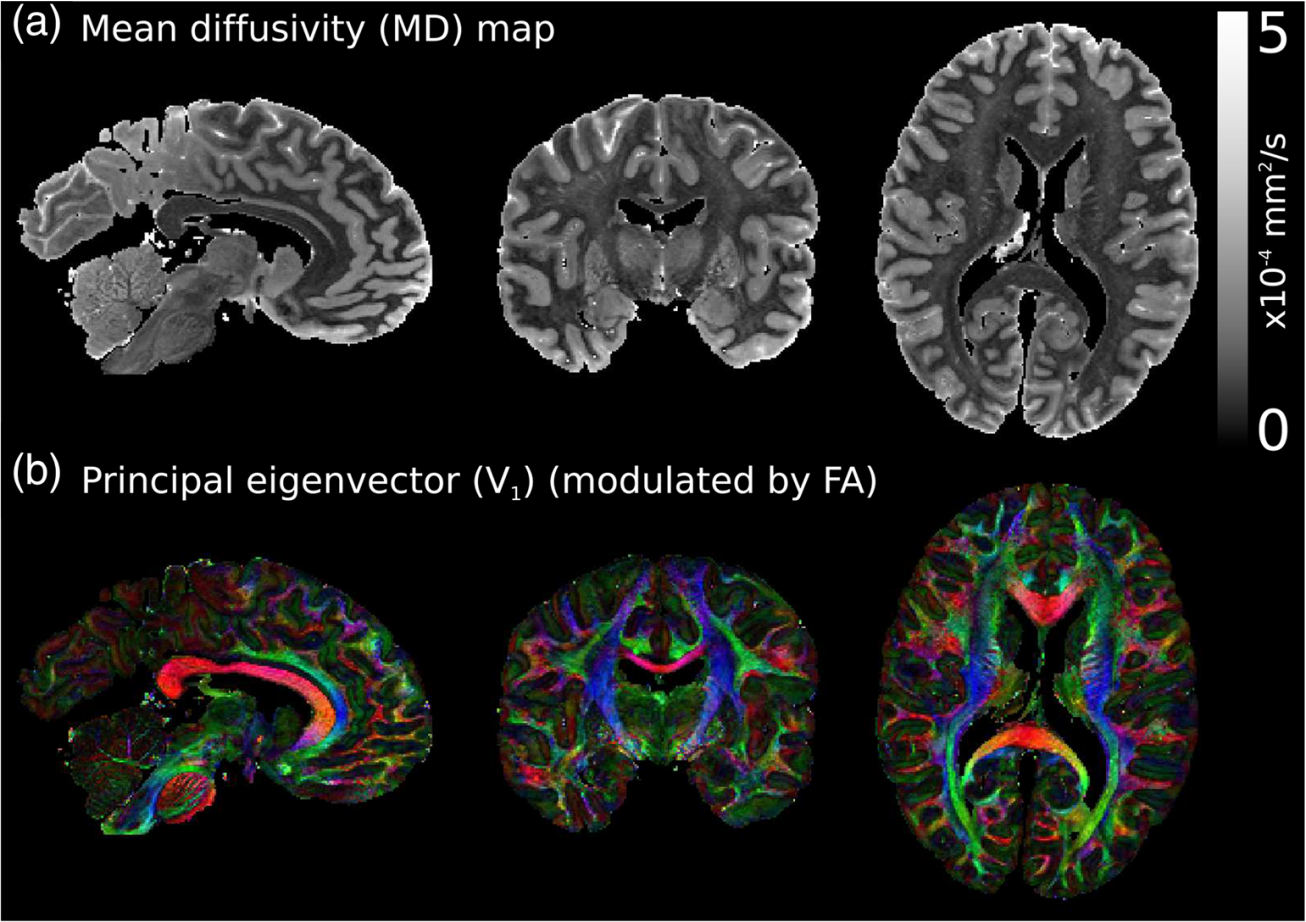
Diffusion tensor estimates in a whole post‐mortem brain. Example diffusion tensor estimates from a single post‐mortem brain used in this study, displaying the (a) mean diffusivity (MD) and (b) principal eigenvector, ${\vec{V}}_{1}$, maps. Both grey and white matter have distinctive diffusivity estimates (a), and diffusion is highly anisotropic across the brain (b). The KT model incorporates these properties when modelling fixative dynamics. ${\vec{V}}_{1}$ maps modulated by the fractional anisotropy (FA), where red: left–right, green: anterior–posterior, blue: superior–inferior. Diffusion imaging and processing protocol for this post‐mortem dataset is described in Tendler et al. (2020)

Previous groups (Dawe et al., 2009; Yong‐Hing, Obenaus, Stryker, Tong, & Sarty, 2005) have investigated the process of fixation by comparing MR estimates in tissue undergoing immersion fixation (influx of fixative) with mathematical models of fixative dynamics. Yong‐Hing, Obenaus, Stryker, Tong, and Sarty (2005) modelled the influx of formalin fixative into a whole, human brain sample undergoing fixation by approximating the brain as a solid sphere and assuming a uniform isotropic diffusion coefficient. Dawe et al. (2009) extended this approach by incorporating the geometry of the brain surface in hemispheres undergoing fixation. Here we build on this previous work by incorporating voxelwise diffusion tensor estimates (derived from diffusion MRI data from the same tissue sample) into our simulations (Figure 1), known as the “kinetic tensor” (KT) model. The KT model assumes that the concentration‐driven diffusion of fixative can be modelled based on the self‐diffusion process of water measured with diffusion MRI. We hypothesise that this will allow for more accurate modelling within different tissue types (e.g., grey and white matter) and incorporation of the orientation dependence of diffusivity estimates (due to diffusion anisotropy).

To achieve this, the concentration of fixative within tissue is simulated using Fick's second law (Harpold, Alvord, & Swanson, 2007; Jbabdi et al., 2005):

(1) $$\frac{\partial c\left(t,\vec{r}\right)}{\partial t}=\nabla \cdot \left(D\left(\vec{r}\right)\nabla c\left(t,\vec{r}\right)\right),$$

where, $c\left(t,\vec{r}\right)$ is the concentration of fixative at time t and position $\vec{r}$, and $D\left(\vec{r}\right)$ is the diffusion tensor:

(2) $$D\left(\vec{r}\right)=\left[\begin{array}{ccc} D_{11}\left(\vec{r}\right) & D_{12}\left(\vec{r}\right) & D_{13}\left(\vec{r}\right)\\ D_{21}\left(\vec{r}\right) & D_{22}\left(\vec{r}\right) & D_{23}\left(\vec{r}\right)\\ D_{31}\left(\vec{r}\right) & D_{32}\left(\vec{r}\right) & D_{33}\left(\vec{r}\right) \end{array}\right],$$

and the diffusion tensor is assumed to be symmetric (i.e., $D_{\mathrm{ij}}\left(\vec{r}\right)=D_{\mathrm{ji}}\left(\vec{r}\right)$). Given a set of initial conditions of the concentration distribution at t=0, Equation (1) can be evaluated. For example, the process of fixation (influx of fixative) can be modelled by assuming starting conditions of 0% fixative concentration within tissue and 100% fixative within the surrounding medium:

(3) $$c\left(0,{\vec{r}}_{\text{tissue}}\right)=0\;\text{and}\;c\left(0,{\vec{r}}_{\text{medium}}\right)=1.$$

For the outflux of fixative (from fully fixed tissue into the surrounding medium), we would have the opposite starting conditions:

(4) $$c\left(0,{\vec{r}}_{\text{tissue}}\right)=1\;\text{and}\;c\left(0,{\vec{r}}_{\text{medium}}\right)=0.$$

Here, we define c between 0 and 1, a unitless fractional concentration of fixative relative to the full concentration of the fixative solution.

#### 2.1.1 Incorporating realistic tissue geometries

Analytical solutions to Equation (1) are only available when assuming simplified tissue geometries (e.g., approximating the brain as a sphere; Yong‐Hing et al., 2005). To incorporate realistic tissue geometries of the brain, Equation (1) must be evaluated using an alternative means. Here we utilise a finite differences approach (as previously described in Jbabdi et al., 2005) to model fixative dynamics within the brain. With finite differences, the spatial distribution of fixative is updated iteratively over a series of n time steps.

**FIGURE 2:**
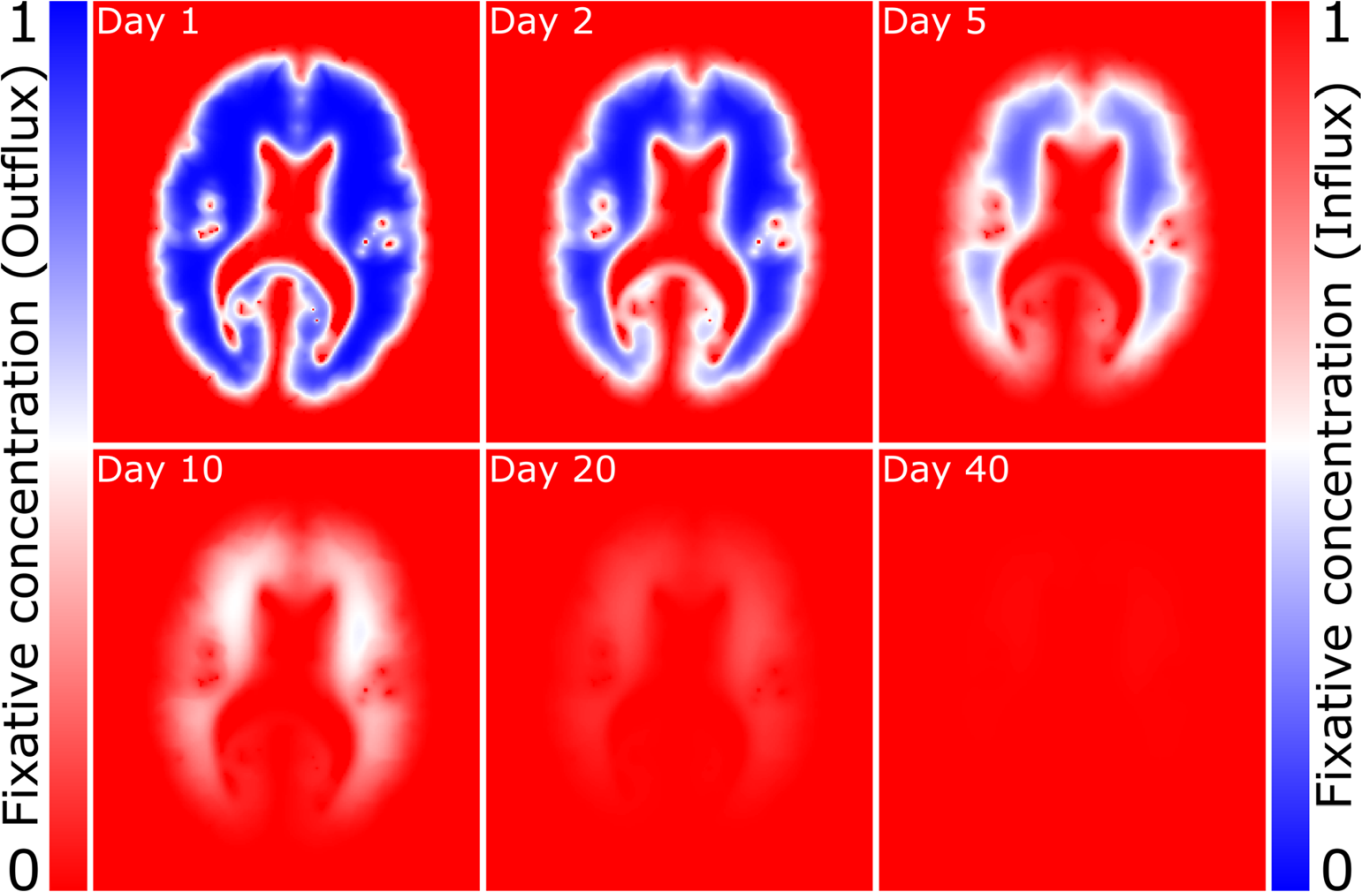
Modelling the influx/outflux of fixative using the KT model. Influx: Defining initial conditions from Equation (3) (0% fixative concentration in tissue surrounded by 100% fixative – right colourbar), the KT model simulates the influx of fixative into tissue, accounting for both the relative diffusion coefficients of different tissue types and diffusion anisotropy (Figure 1). Over time, fixative penetrates further into the brain, eventually leading to fully fixed tissue. For this brain sample, all voxels had a fixative concentration >0.99 after 46 days, in broad agreement with a previous experimental observation reporting formalin fixation within approximately 38 days in a whole, human brain (Yong‐Hing et al., 2005). Outflux: Defining initial conditions from Equation (4) (100% fixative concentration in tissue surrounded by 0% fixative – left colourbar), the fixative outflux simulation is equal to the complement of the influx simulation (i.e., 1 – influx). Over time the fixative concentration reduces throughout the brain, eventually leading to tissue with no fixative solution remaining. KT model simulation performed using the diffusion tensor estimates in Figure 1. Concentrations defined between 0 and 1, where 1 corresponds to a voxel containing 100% fixative

To achieve this, Equation (1) is discretized and rearranged to solve for concentration $c\left(t,\vec{r}\right)$ [Equation (A1)]. The spatial distribution of fixative concentration is subsequently simulated over a series of n time steps given a set of initial conditions [e.g., Equations (3) and (4)]. Each time step estimates the change in concentration over the time period τ=T/n, where T is the total duration of the simulation. Figure 2 illustrates the simulated dynamics of fixative influx [initial conditions defined by Equation (3)] into a whole post‐mortem brain using the finite difference approach and the KT model. Fixative initially penetrates into the brain tissue through surfaces in contact with the fixative solution. Over time fixative moves further into the tissue, eventually leading to c=1 across the entire brain.

#### 2.1.2 Kinetic tensor based confound regression

Prior to scanning, brain samples are often removed from fixative and transferred into an alternative fluid that has more favourable imaging properties. This will lead to a concentration boundary at the brain surface [initial conditions defined by Equation (4)], which may drive the outflux of fixative into the surrounding medium. Any outflux of fixative will lead to a decrease in its concentration in tissue and therefore a change in T_2_. When considering formalin, a previous study has estimated a 10–15 ms linear decrease in T_2_ per 2% concentration (Shepherd, Thelwall, et al., 2009a).

Figure 2 presents a simulation of the reduction in fixative concentration after modelling outflux [initial conditions defined by Equation (4)] in a whole, post‐mortem brain using the KT model. Initially, a reduced concentration of fixative is predicted within brain regions in close proximity to the brain surface, eventually leading to complete removal of fixative within the brain after approximately 40 days. Large changes in concentration are observed near the brain surface within the first 2 days of immersion.

We propose using the resulting fixative concentration map derived from simulation as a confound regressor to account for the effect of fixative concentration on the quantitative T_2_ map. We perform this correction with the assumption that T_2_ varies linearly with fixative concentration (Shepherd, Thelwall, et al., 2009a), defining:

(5) $$T_{2}=T_{2_{0\%}}+\beta \cdot c,$$

where, $T_{2_{0\%}}$ is the T_2_ estimated at 0% fixative concentration and β describes the rate of change of T_2_ with fixative concentration. Here we perform this correction as a global regression, estimating a single $T_{2_{0\%}}$ and β per brain. Using the estimate of β, we can subsequently perform a voxelwise regression of the fixative concentration to generate a $T_{2_{0\%}}$ map; that is, the predicted T_2_ map in the absence of fixative.

We compare this “*kinetic tensor*” (KT) correction to similar global regressions based on two other models: (i) a “*kinetic isotropy*” (KI) correction that assumes isotropic diffusivities, and (ii) a “*distance‐to‐surface*” (D2S) correction that considers only how close each voxel is to the nearest surface. The D2S model is a phenomenological correction that does not model fixative per se, but captures a simple geometric feature that relates to the flux of fixative.

## 3 METHODS

### 3.1 Data acquisition and processing

Fourteen whole, formalin‐fixed, post‐mortem brains (consisting of 11 brains from patients diagnosed with amyotrophic lateral sclerosis and 3 controls) were used in our experiment. Post‐mortem brains were extracted from the skull and immersion fixed in formalin (mean post‐mortem delay = 3.1 ± 1.4 days, minimum = 1 day, maximum = 7 days). All brains were formalin‐fixed for at least 1 month (mean duration = 116 ± 64 days, minimum = 35 days, maximum = 283 days) prior to scanning. Of these 14 brains, seven were fixed in 10% formalin and seven were fixed in 10% neutral‐buffered formalin (NBF). The 10% formalin solution was made in‐house by diluting 40% formaldehyde (Genta Medical, UK) in water (neutralised using marble chips). The 10% NBF solution (Genta Medical, UK) consisted of formaldehyde diluted in phosphate buffered saline (PBS). Details of individual brains are provided in Table S1. The study was conducted under the Oxford Brain Bank's generic Research Ethics Committee approval (15/SC/0639).

Prior to scanning, excess formalin was removed from the brain surface and drained from the ventricles. Brains were subsequently submerged in fluorinert (3M‐FC‐3283), a perfluorocarbon‐based fluid that generates no MR signal and is susceptibility matched to tissue, used to improve imaging quality. After filling of the ventricles with fluorinert and manual manipulation to remove air bubbles, brains were placed inside a custom made scanning container filled with fluorinert. Full details of the brain packing process are given in Wang et al. (2020). All brains were immersed in fluorinert for approximately 48 hr prior to scanning.

Brains were scanned on a 7 T whole body Siemens system using a 32‐channel receive/1‐channel transmit head coil (Nova Medical). For estimating T_2_ maps, we used a multiecho turbo spin‐echo (TSE) sequence with 6 echoes, (TE = 13, 25, 38, 50, 63, 76 ms, where each TE was obtained in a separate acquisition) and additional parameters: TR = 1,000 ms, resolution = 0.9 × 0.9 × 0.9 mm^3^, bandwidth = 166 Hz/pixel, turbo factor = 6, time per acquisition = 36 min. These represent the typical imaging parameters for our T_2_ imaging protocol; the exact parameters evolved over the time‐course of our experiment. Full details of the parameters for each sample are provided in Table S2. To account for any small changes in brain position between TEs, coregistration was performed using FSL FLIRT (Jenkinson & Smith, 2001; 6 degrees of freedom transformation), though this typically led to no observable change in the resulting images.

When performing T_2_ mapping, B_1_ inhomogeneity can cause the signal to deviate from mono‐exponential decay due to incomplete refocusing of echoes. As our 7 T data was observed to demonstrate this effect, quantitative T_2_ maps were derived through voxelwise fitting of the signal using an extended phase graph (EPG) model that includes estimates of the B_1_ profile (Hennig, 1991a, 1991b; Weigel, 2015). Full details of our EPG fitting implementation is provided in Supporting information S1, with associated code available at https://github.com/BenjaminTendler/KT_model. This code is based on EPG software (Weigel, 2015) available at https://github.com/matthias‐weigel/EPG.

While one might base KT modelling on a diffusion tensor multisubject atlas, in this case we have access to diffusion MRI for each individual brain sample being studied. Diffusion MRI data were acquired in each post‐mortem brain using a diffusion‐weighted steady‐state free precession (DW‐SSFP) sequence (Foxley et al., 2014; Kaiser, Bartholdi, & Ernst, 1974; Le Bihan, 1988; Merboldt et al., 1989a; Merboldt, Hänicke, Gyngell, Frahm, & Bruhn, 1989b), from which diffusion‐tensor estimates (three eigenvectors, ${\vec{V}}_{1,2,3}$, and three eigenvalues, $L_{1,2,3}$) were derived over the whole brain at an effective *b*‐value (*b*_eff_) of 4,000 s/mm^2^. Details of the full acquisition protocol and processing pipeline for the diffusion data to a single *b*_eff_ are described in Tendler et al. (2020), and the full post‐mortem protocol is described in Pallebage‐Gamarallage et al. (2018). Example diffusion tensor estimates for a single post‐mortem brain used in this study are displayed in Figure 1.

### 3.2 Modelling the outflux of fixative

The concentration of fixative within each brain was simulated assuming outflux into the surrounding medium for 48 hr (2000 time steps, τ = 86.4 s) using Equation (A1), with initial conditions as defined in Equation (4). This corresponds to the condition where a fully fixed brain $c\left(0,{\vec{r}}_{\text{tissue}}\right)=1$ is surrounded by a medium with no fixative ($c\left(t,{\vec{r}}_{\text{medium}}\right)=0$), consistent with the long tissue fixation periods in this study (Table S1). Throughout the simulation the concentration of fixative in the surrounding medium was kept constant [$c\left(t,{\vec{r}}_{\text{medium}}\right)=0$]. Although experimentally outflux will lead to a small concentration of fixative in the surrounding medium, given the time frame of our experiment (48 hr) and the volume of the surrounding medium used in our experiments, we expect this to be a reasonable assumption. To prevent artefacts in the resulting simulations, voxels with spuriously high diffusion coefficients (empirically determined as >1 × 10^−3^ mm^2^/s) were set equal to the mean of the surrounding tissue. Full details surrounding this correction are provided in Supporting information S1.

For the KT model, voxelwise diffusion tensors ($D\left(\vec{r}\right)$) estimated over each post‐mortem brain (Figure 1) were fed into Equation (A1). To assess the importance of incorporating diffusion anisotropy and voxelwise diffusion coefficients, two alternative models were investigated:

1. The KI model, which assumes an isotropic uniform diffusion throughout tissue. Here, the diffusion tensor ($D\left(\vec{r}\right)$) in Equation (2) is substituted with a diagonal matrix, with each diagonal component set to the average mean diffusivity over the entire post‐mortem brain. Only a single diffusion coefficient estimate and tissue mask are required to simulate the KI model.
2. The D2S model, which assumes the concentration of fixative in any given voxel is proportional to its distance (in mm) to the nearest surface. This model assumes a simple linear relationship between fixative concentration and the distance to surface (as opposed to accounting for fixative dynamics within tissue). The D2S model does not perform diffusion‐based modelling, and is therefore independent of the diffusion coefficient of tissue. It was calculated using the distancemap function in FSL (Smith et al., 2006).

Code for the KI and KT model used in this study is available at https://github.com/BenjaminTendler/KT_model.

### 3.3 Fixative correction

The simulated fixative concentration maps were removed as a confound from our T_2_ maps by first fitting with Equation (5). Fitting was performed as a single global regression, estimating a single value of $T_{2_{0\%}}$ and β per brain. The estimated β was subsequently used to perform a voxelwise regression across the brain, to determine a voxelwise $T_{2_{0\%}}$ estimate (described further below). One concern in fitting is potential tissue‐type bias. Grey and white matter tissue are characterised by different T_2_ values and exhibit a spatial pattern that varies from centre to periphery (Figure S5). Hence, it is likely that the true T_2_ maps will to some degree correlate with fixative models, since they share this general spatial distribution. To eliminate tissue‐type bias on our fitting, $T_{2_{0\%}}$ and β were estimated for a given brain from white matter voxels only. White matter masks were generated using FSL FAST (Zhang, Brady, & Smith, 2001) from the L_3_ diffusion tensor estimates. Both the concentration maps and tissue masks were estimated in the diffusion space of the post‐mortem brains, and transformed to the space of the T_2_ maps using FSL FLIRT (Jenkinson & Smith, 2001; 6 degrees of freedom, estimated from the unprocessed TSE and DW‐SSFP b0 data). A 6 degrees of freedom transformation was sufficient as the acquisition bandwidth of the diffusion scans (393 Hz/Pixel; Tendler et al., 2020) was similar to the TSE scans (166 Hz/Pixel).

To perform the fitting, T_2_ estimates from all white matter voxels were binned according to concentration (100 bins, range 0–1 for the KI and KT models, 0–23 mm for the D2S model), and the mean T_2_ estimated for each bin. Outliers (T_2_ estimates greater/less than the median ± 3 × median absolute deviation across all white matter) were not included in this calculation (and will not be included when presenting results in this manuscript). In very close proximity to the brain surface, T_2_ values were higher and characterised by a larger T_2_ error in comparison to other tissue. To avoid these boundary effects, voxels within 2 mm to the brain surface were additionally not included in these calculations. The binned data across the concentration range was fit to Equation (5), with the fitting weighted by the number of voxels per bin.

The voxelwise influence of fixative concentration was subsequently eliminated over the entire brain to generate $T_{2_{0\%}}\left(x,y,z\right)$ maps via:

(6) $$T_{2_{0\%}}\left(x,y,z\right)=T_{2}\left(x,y,z\right)-\beta \cdot c\left(x,y,z\right),$$

where, *β* is a single brain‐wide scalar and $T_{2_{0\%}}\left(x,y,z\right)$ is a spatial map providing a voxelwise estimate of $T_{2_{0\%}}$ [as opposed to the scalar $T_{2_{0\%}}$ required to estimate β in Equation (5)]. For the D2S model, $c\left(x,y,z\right)$ is substituted for the distance to surface measurement.

The motivation behind performing fixative concentration correction across both grey and white matter using $T_{2_{0\%}}$ and β estimates derived from white matter voxels only is two‐fold. First, if the outflow of fixative is a diffusion driven process, the relationship between T_2_ and fixative concentration should be driven by the diffusivity properties of tissue only, as opposed to other tissue‐specific properties. Conservatively, we therefore expect there to be no difference in the relationship between T_2_ and fixative concentration for both grey and white matter. Second, this provides the opportunity to validate our approach based on a tissue type that our model has not seen (grey matter). An observed improvement in homogeneity for grey matter voxels based on a white matter correction would not be a trivial result, validating our expectation that fixative outflow relates to the diffusivity properties of our samples.

In the absence of a ground truth, we require a metric for comparing across models. Although T_2_ is likely to vary across the brain within a given tissue type, spatial patterns matching a spatial model of fixative concentration should most conservatively be attributed to fixative. The fact that a single regression coefficient was fit to all of white matter means that it is unlikely to result in over‐fitting. Performance of the different models was thus evaluated by comparing the homogeneity of the T_2_ maps before and after correction within tissue type. A concentration model is deemed to be “better” if it improves homogeneity (i.e., if it removes more variance) compared with another model. Importantly, this correction is motivated by the previous observation that T_2_ has a linear dependency on fixative concentration (Shepherd, Thelwall, et al., 2009a). Therefore, as only this component is modelled and removed when we perform our correction [Equations (5) and (6)], an increase in homogeneity corresponds to an improved identification and elimination of the linear fixative concentration confound.

### 3.4 Correlation with ferritin and PLP

This work forms part of a larger post‐mortem imaging project investigating how changes in MR image contrast due to the neurodegenerative disease amyotrophic lateral sclerosis (ALS) relate to pathology as reflected in histological staining (Pallebage‐Gamarallage et al., 2018). As part of this project, immunohistochemical staining has been performed within each brain for ferritin (an iron storage protein and a non‐quantitative surrogate for iron content in tissue) and PLP (a major myelin protein). Tissue sections with these stains have been acquired in the primary motor cortex (M1), secondary visual cortex (V2) and anterior cingulate cortex (ACC). Full details of the histology acquisition and processing pipeline are provided in Pallebage‐Gamarallage et al. (2018). We assess the correlation between the transverse relaxation rate *R*_2_ and ferritin/PLP with and without correction for fixative concentration, based on the expectation of a linear relationship between *R*_2_ and, for example, ferritin content (Vymazal et al., 1992).

PLP and ferritin are quantified using stained area fraction (SAF). The SAF is defined as the ratio of the positively stained region of the analysed region of interest (ROI) relative to the total ROI. In this study, PLP SAF estimates are available for both hemispheres of M1 (in the leg, hand and face areas), V2 and the ACC. For ferritin, SAF estimates are available in the left hemisphere only for M1 (leg and face regions), V2 and the ACC. Ferritin staining was performed in two separate batches (batch 1 – M1 leg, V2 and ACC; batch 2 – M1 face, V2 and ACC). To account for cross‐batch variability, each batch was normalised (demeaned and divided by the *SD*) prior to combining the two batches, with normalisation performed separately for brains fixed with 10% NBF and 10% formalin. To make comparisons with the *R*_2_ estimates, ROIs were generated in the diffusion space of the MRI data in the left and right hemispheres of M1, V2 and the ACC. For the motor cortex, standard space label masks were coregistered into the space of the post‐mortem brains using FLIRT (Jenkinson & Smith, 2001), followed by manual segmentation into leg, hand and face areas of the motor cortex. For V2 and ACC, masks were hand drawn in the space of the diffusion MRI data using the histology images as a guide, where the diffusion MRI data was chosen due to its strong grey‐white matter contrast. All masks were generated by a researcher familiar with neuroanatomy. Masks were subsequently coregistered into the space of the T_2_ maps using FLIRT (Jenkinson & Smith, 2001). Any white matter areas were removed from the resulting masks prior to analysis. T_2_ estimates was taken as the median value over the ROI, with the reciprocal taken to estimate *R*_2_.

## 4 RESULTS

**FIGURE 3:**
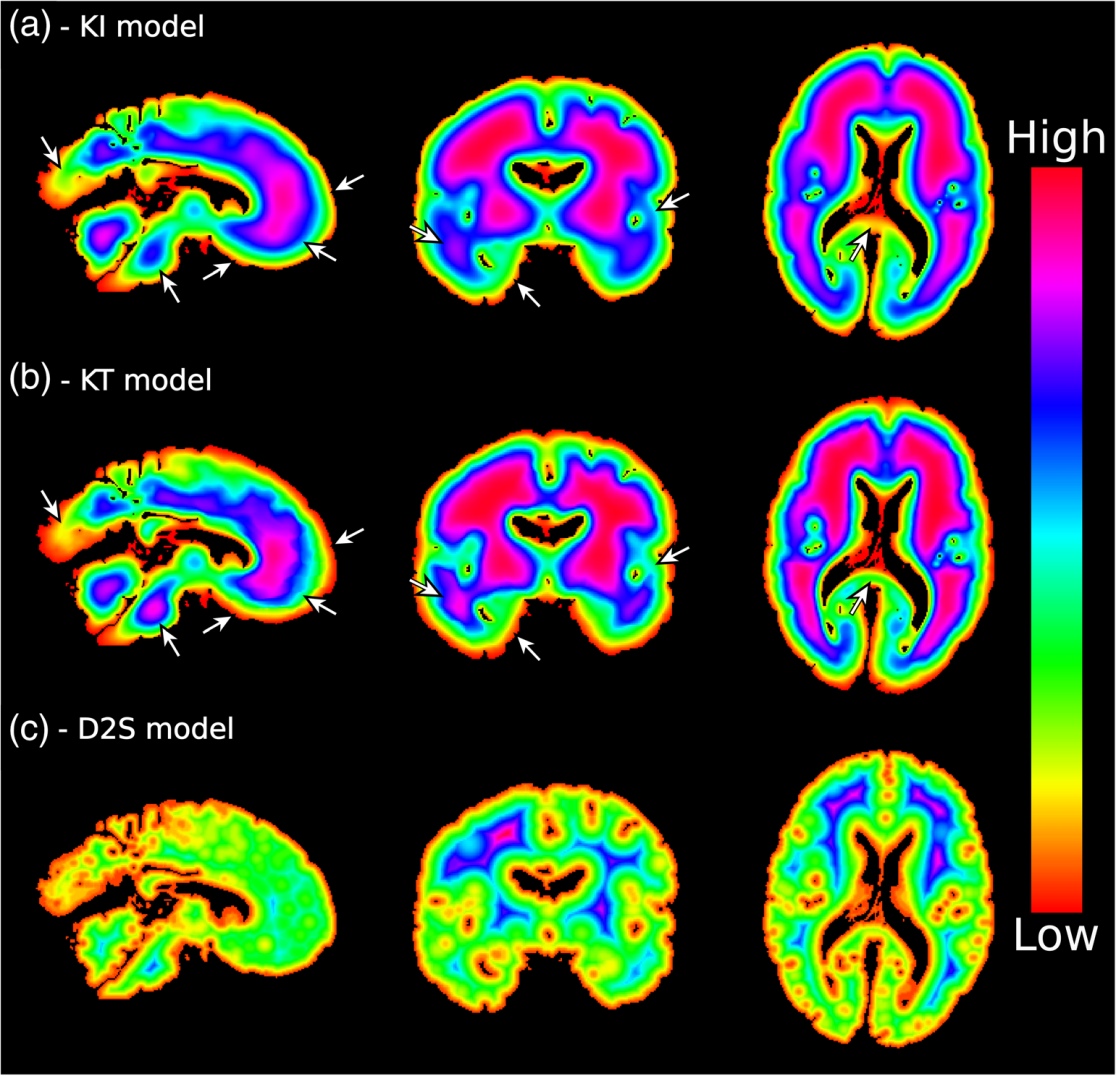
Modelling the outflux of fixative with the KI, KT and D2S model. Defining initial conditions from Equation (4) (100% fixative concentration in tissue surrounded by an external medium of 0% fixative), here we display the resulting concentration distribution map for the kinetic isotropy (a) and kinetic tensor (b) models, and the phenomenological distance‐to‐surface (c) model. Subtle differences between the KI (a) and KT (b) models are apparent across both grey and white matter (white arrows). The D2S model (c) reveals a considerably different distribution across the brain. (a,b) modelled using the diffusion tensor estimates in Figure 1 assuming fixative outflux for 48 hr. (a,b) are scaled between 0 and 1, with (c) scaled between 0 and 19.5 mm. Colormap chosen to highlight the differences across the brain

**FIGURE 4:**
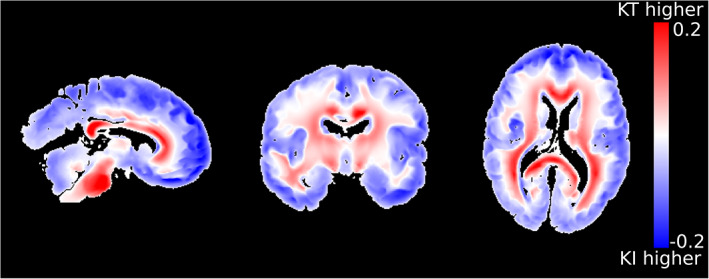
Differences between the KI and KT model. By examining the concentration difference between the KT and KI models (KT model minus KI model), it is apparent that the KT model is characterised by an increased fixative concentration across white matter, with a decreased concentration across grey matter versus KI. This is consistent with observations of an increased/decreased diffusion coefficient across grey/white matter in post‐mortem brains (Figure 1a) versus the mean diffusivity. Figure formed from the data in Figure 3. Concentration distributions modelled using the diffusion tensor estimates in Figure 1 assuming fixative outflux for 48 hr

Figure 3 displays the simulated outflux of fixative using the KI and KT models, alongside the phenomenological D2S model in a single brain sample. Whereas the D2S model (Figure 3c) reveals a markedly different distribution across the brain, relatively subtle differences are observed between the KI (Figure 3a) and KT (Figure 3b) models. By taking the difference between these two maps (Figure 4), it becomes apparent that the KT model exhibits an increased concentration of fixative in white matter and a decreased concentration of fixative in grey matter. This observation is consistent with the diffusion coefficients used for the two models. Notably, post‐mortem tissue is characterised by higher diffusivity in grey matter (average *D* = 3.1 ± 1.5 × 10^−4^ mm^2^/s across all brains) versus white matter (*D* = 1.37 ± 0.39 × 10^−4^ mm^2^/s; Figure 1a). The average measured diffusivity across the whole brain (*D* = 2.4 ± 1.5 × 10^−4^ mm^2^/s) used in the KI model is higher than the tissue diffusivity in white matter, but lower than grey matter. This leads to decreased fixative outflow in white matter for the KT model versus the KI model, and opposite for grey matter, which drives the observation of increased/decreased concentration of fixative in white/grey matter, respectively.

**FIGURE 5:**
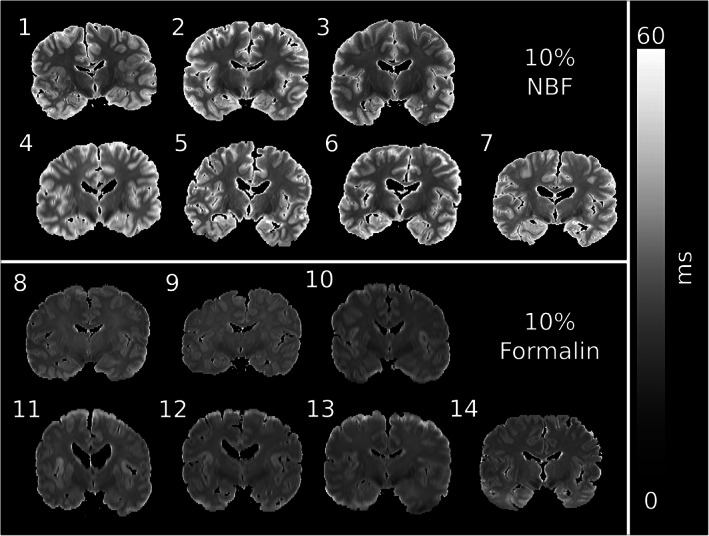
Single coronal slice of the T_2_ maps from all 14 brains. Our EPG framework (details provided in Supporting information S1) accounts for the influence of B_1_ homogeneity at 7 T, reducing the bias on T_2_ estimates in areas of low B_1_. Brains fixed with 10% NBF display significantly higher T_2_ estimates in both white and grey matter (see Figure 6)

**FIGURE 6:**
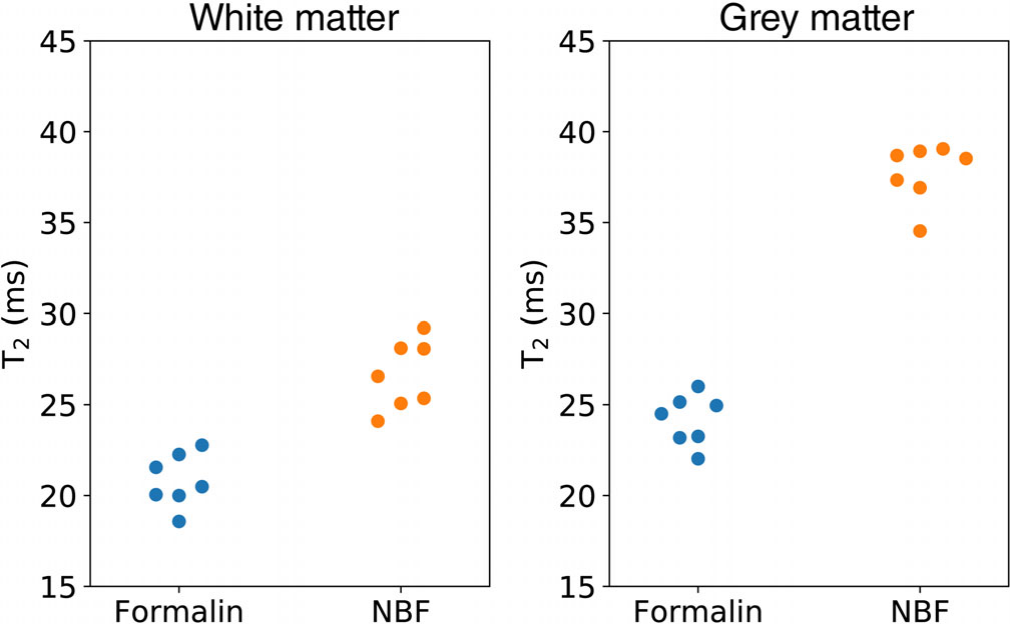
Mean T_2_ for brains fixed with 10% formalin and 10% NBF over white and grey matter. Brains fixed with 10% NBF were characterised by a higher estimate of T_2_ over white matter (*p* = 4.2 × 10^−5^, Cohen's *D* = 3.2) and grey matter (*p* = 1.4 × 10^−9^, Cohen's *D* = 8.3), with differences clearly depicted in Figure 5. Each dot represents the mean T_2_ over white/grey matter for a single brain. The *p*‐values estimated using Welch's *t*‐test. Horizontal displacement along x‐axis for individual points is for visualisation purposes only

Figure 5 displays a single coronal slice of the T_2_ maps from all 14 brains, demonstrating that our EPG framework (details provided in Supporting information  S1) generates T_2_ maps that exhibit consistent contrast across grey and white matter for each fixative type. Figure 5 additionally reveals that the fixative type has a considerable effect on the magnitude of T_2_ estimates, with an increased T_2_ observed in both white and grey matter (Figure 6) for brains fixed with 10% NBF versus 10% formalin. No significant associations were found between the mean T_2_ across the entire brain and the post‐mortem delay/time in fixative before scanning (values provided in Table S1) for either fixative type.

**FIGURE 7:**
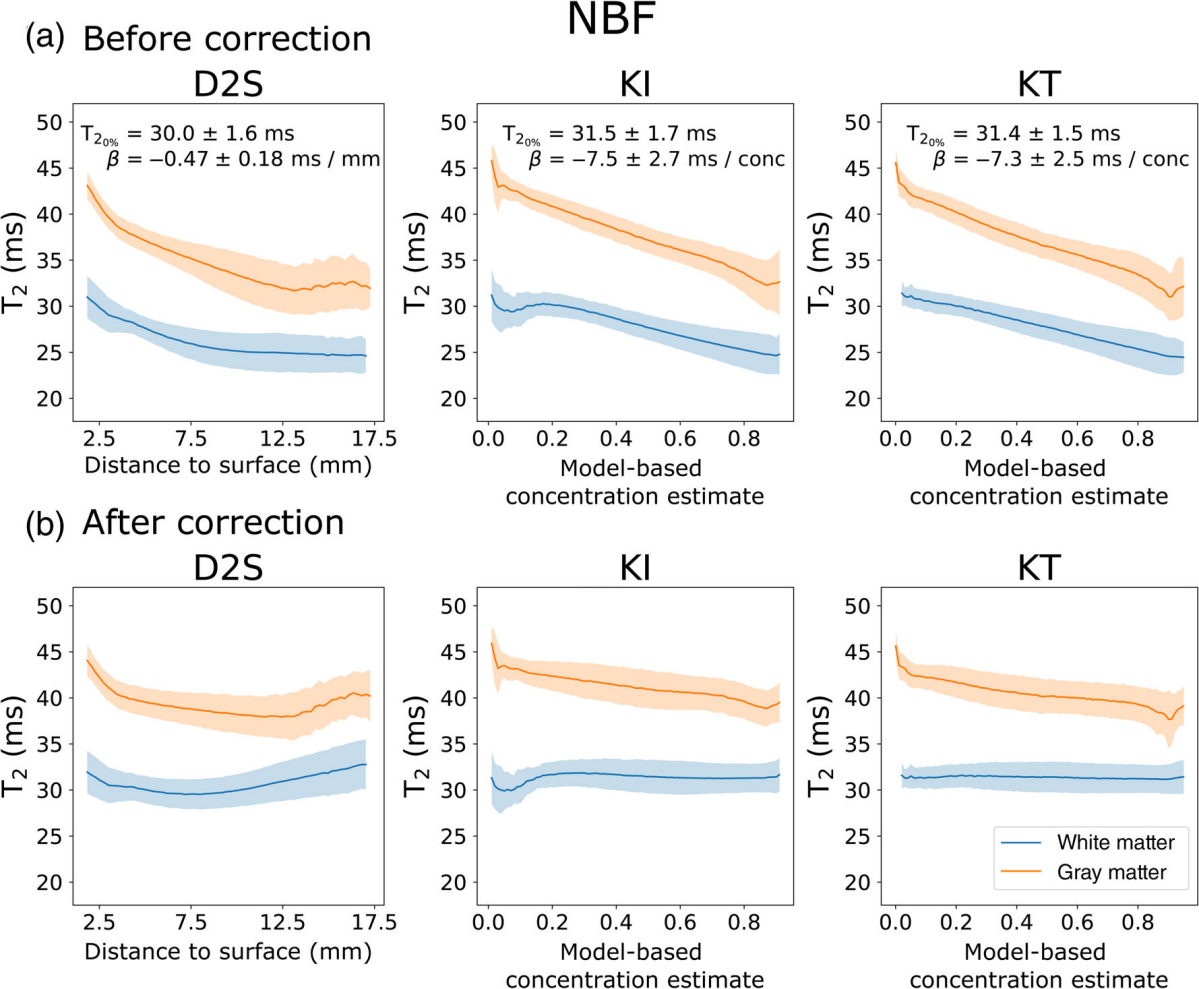
T_2_ versus concentration/distance to surface over white and grey matter for all post‐mortem brains fixed with 10% NBF. Averaging over all brains fixed with 10% NBF, all three models display a decrease in T_2_ with increased concentration/distance to surface (a). Whereas the KI and KT models demonstrate a linear relationship (in agreement with Shepherd, Thelwall, Stanisz, & Blackband, 2009a), the D2S model displays a more inhomogeneous change in T_2_. Regressing out the influence of fixative concentration using Equation (6) improves the homogeneity of T_2_ estimates across white and grey matter in all three models (b). Results displayed as the mean ± *SD* across all brains fixed with 10% NBF

**FIGURE 8:**
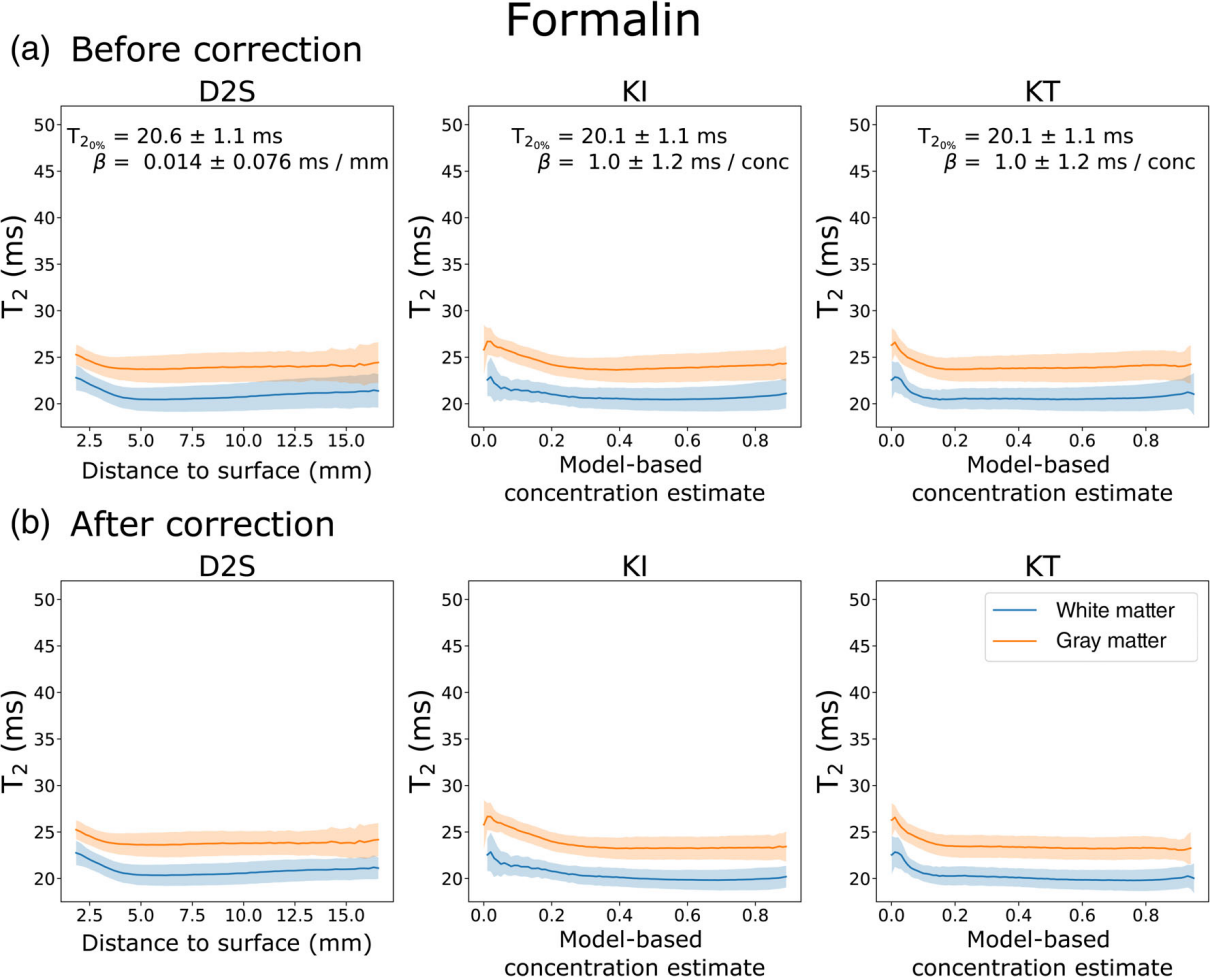
T_2_ versus concentration/distance to surface over white and grey matter for all post‐mortem brains fixed with 10% formalin. Averaging over all brains fixed with 10% formalin, all three models display a small decrease in T_2_ with increased concentration/distance to surface (a). This change is inhomogeneous across all three models, where the change in T_2_ is characterised by a small β for all three models. Regressing out the influence of fixative concentration using Equation (6) leads to little apparent change across white and grey matter in all three models (b). Results displayed as the mean ± *SD* across all brains fixed with 10% formalin

Figure 7 displays the relationship of T_2_ versus concentration across white and grey matter using the D2s, KI, and the KT models for brains fixed with 10% NBF. In all cases, the model appears to explain a large amount of variation in T_2_. An approximately linear decrease in T_2_ with increases in fixative concentration is apparent for the KI and KT models prior to correction (Figure 7a), in agreement with previous reports (Shepherd, Thelwall, et al., 2009a). The D2S model similarly displays a decrease in T_2_ with increased distance to surface, but is more inhomogeneous across the distance profile. In addition, the binning of voxels according to the D2S model results in higher SD, suggesting that distance to surface is less relevant to predicting a voxel's T_2_ than the KI and KT concentration models. By correcting for the influence of fixative concentration using Equation (6) (Figure 7b), all three models produce visibly flatter profiles across a wide range of concentrations in white matter (i.e., voxels included in the fit), and reduce the inhomogeneity across grey matter (i.e., voxels not include in the fit). The KI and KT models produced notably flatter profiles compared with D2S. Interestingly, brains fixed with formalin did not show the same trend, with changes in T_2_ on the order of a few ms over the entire concentration range (Figure 8). Correction across these samples led to little observable change for all three models.

**FIGURE 9:**
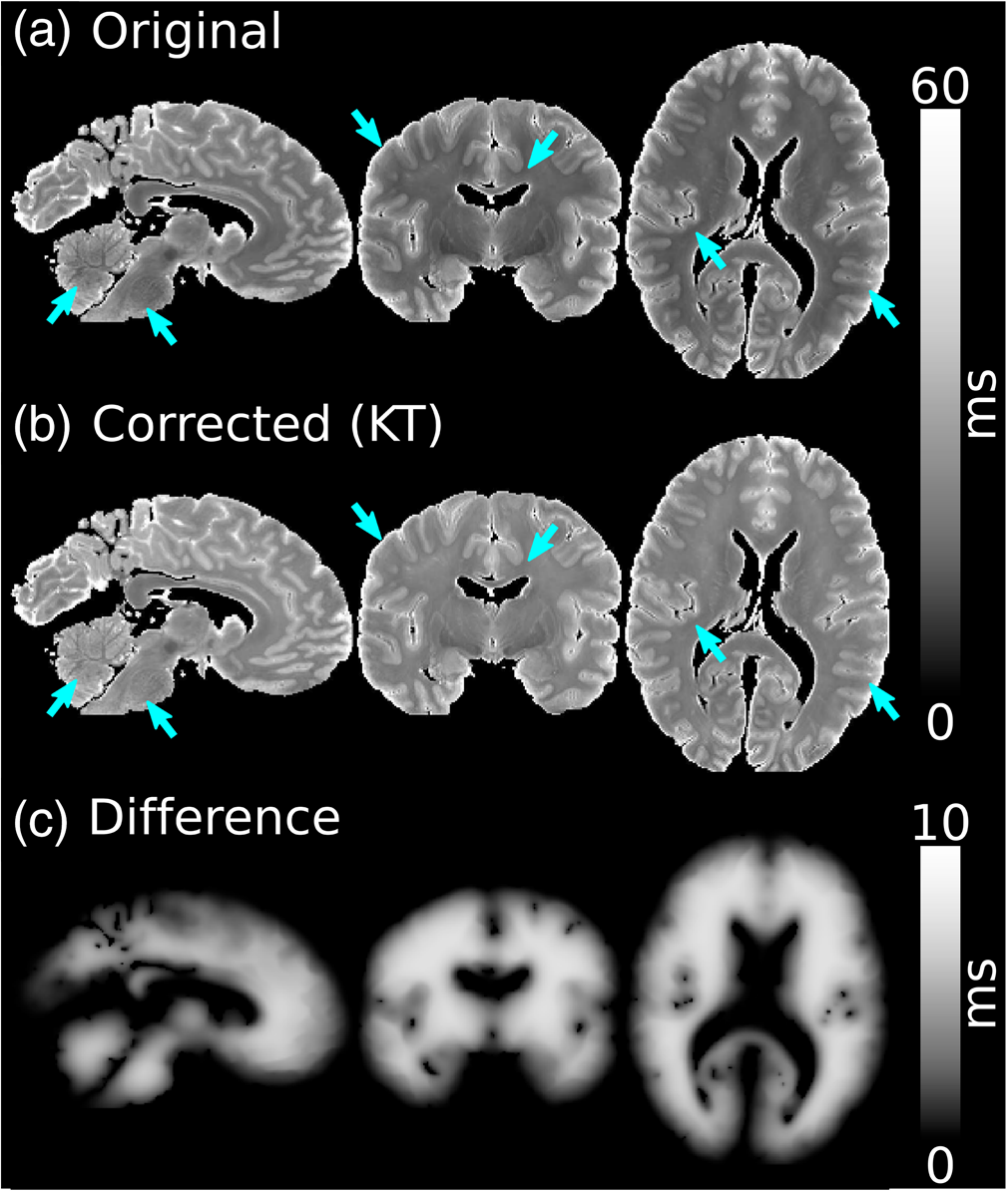
T_2_ map before and after correction with the KT model. By performing a correction with the KT model over a post‐mortem T_2_ map (a), we are able to reduce the inhomogeneity across the brain (b). These differences are most apparent within regions close to the brain surface (a and b arrows). The difference map (c – corrected minus original) is a scaled KT concentration distribution

Table 1 displays the inhomogeneity (defined here in terms of the *SD*) across brains fixed with 10% NBF (Table 1a) and 10% formalin (Table 1b) within grey and white matter separately before and after correction. T_2_ maps for brains fixed with 10% NBF are characterised by a higher inhomogeneity across both white and grey matter prior to correction. In these brains (Table 1a), corrections based on all three models reduced inhomogeneity. Notably, this improvement is observed for both white and grey matter, despite the model being fit to white matter voxels only. The KI and KT models reveal similar performance, with the KT model demonstrating the best overall improvement (lowest inhomogeneity over both white and grey matter). Across white matter, the reduction in inhomogeneity reaches significance (defined as *p* < .05) for the KI and KT models. Across grey matter, the KT model demonstrates the best overall reduction in inhomogeneity, although it does not quite reach significance (*p* = .052). Figure 9 displays a 10% NBF brain before and after correction, demonstrating a visible reduction in inhomogeneity across the brain.

**TABLE 1:** Inhomogeneity across white and grey matter for brains fixed with 10% NBF and 10% formalin

(a) 10% NBF				
Tissue type	Uncorrected	D2S correction	KI correction	KT correction
White matter	2.80 ± 0.41	2.48 ± 0.28 (0.12)	2.25 ± 0.17 (0.0068)	2.15 ± 0.17 (0.0022)
Grey matter	6.11 ± 0.77	5.59 ± 0.55 (0.17)	5.44 ± 0.52 (0.083)	5.36 ± 0.50 (0.052)

(b) 10% formalin				
Tissue type	Uncorrected	D2S	KI	KT
White matter	1.46 ± 0.23	1.45 ± 0.22 (0.89)	1.45 ± 0.22 (0.92)	1.44 ± 0.22 (0.87)
Grey matter	2.45 ± 0.32	2.45 ± 0.32 (0.99)	2.47 ± 0.33 (0.91)	2.47 ± 0.33 (0.90)

*Note*: For brains fixed with 10% NBF (a), all three models lead to a reduction in inhomogeneity (defined here as the SD) across the brain. The KI and KT models generate a reduced inhomogeneity across both white and grey matter versus the D2S model. The KI and KT models perform similarly, with the KT model demonstrating the best overall improvement. For brains fixed with 10% formalin (b), all three models lead to very little change in inhomogeneity, notably an insignificant increase in inhomogeneity with the KI/KT models across grey matter. The *p*‐values comparing the change in inhomogeneity for each correction displayed in brackets.

For brains fixed with 10% formalin, none of the corrections lead to much difference in inhomogeneity across both white and grey matter (Table 1b), characterised by very small changes, which do not reach significance. In these brains, the KI and KT models lead to a small increase in inhomogeneity across grey matter (which is possible given that the regression parameters are estimated using white matter only).

**FIGURE 10:**
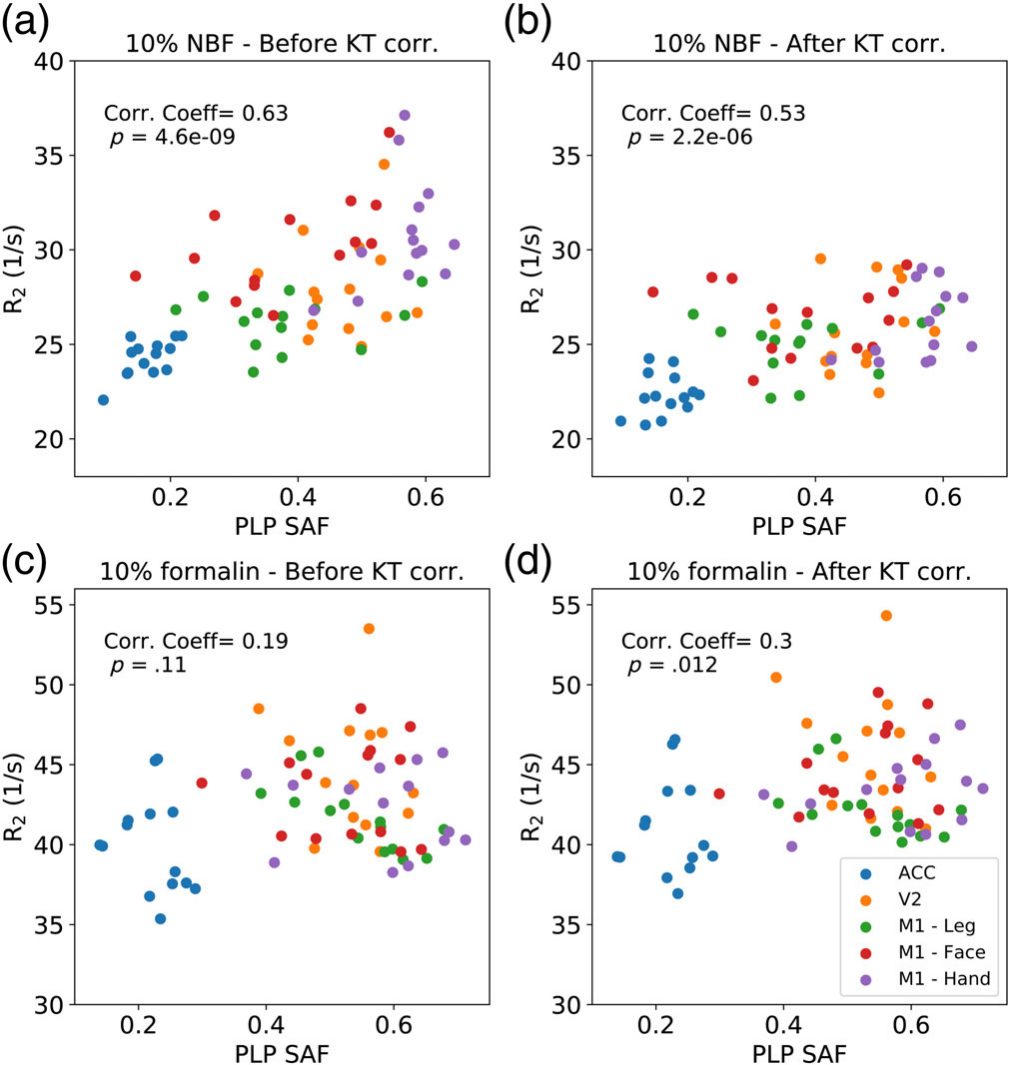
Correlation between *R*_2_ and PLP for brains fixed with 10% NBF and 10% formalin. Brains fixed with 10% NBF (a) and 10% formalin (c) demonstrate a positive correlation with PLP, with the relationship predominantly driven by regional differences in PLP and *R*_2_ across the ROIs used in this study. Correction with the KT model (b and d) improved the similarity of the relationship between the two fixative types, corresponding to a reduced/increased correlation for brains fixed with 10% NBF/10% formalin, respectively

**FIGURE 11:**
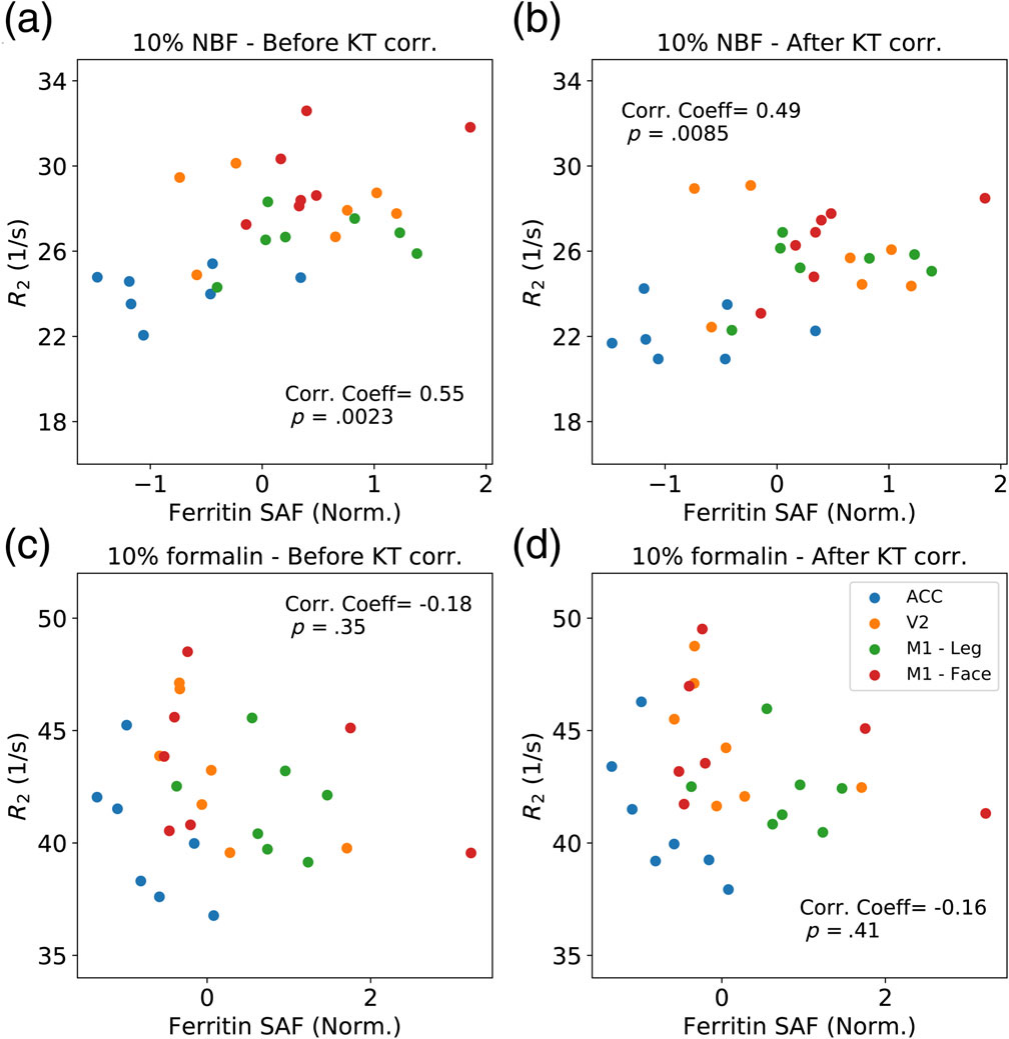
Correlation between *R*_2_ and ferritin for brains fixed with 10% NBF and 10% formalin. Brains fixed with 10% NBF (a) display a positive correlation with ferritin, with a small reduction in the correlation coefficient after KT correction. For brains fixed with 10% formalin, no correlation was observed before (c) or after (d) the KT correction. Note that as the ferritin SAFs were normalised for the two batches, the SAF values can be positive & negative and are not restricted to a range between 0 and 1. As the ACC and V2 regions were included in both batches, the ferritin SAFs were averaged prior to analysis and plotting

Comparisons with histology reveal a positive correlation between the relaxation rate *R*_2_ (1/T_2_) and PLP for both the 10% NBF and 10% formalin brains (Figure 10). Brains fixed with 10% NBF demonstrate a stronger positive correlation than those fixed with 10% formalin, with the correlation for brains fixed with 10% formalin just below significance. Correction with the KT model increased the similarity between the two fixative types, with a small decrease in the correlation between *R*_2_ and PLP for brains fixed with 10% NBF, and a small increase for brains fixed with 10% formalin (reaching significance after correction). For the ferritin results (Figure 11), a positive correlation with *R*_2_ was found for brains fixed with 10% NBF, with a small decrease after correction with the KT model. No significant correlation was found for brains fixed with 10% formalin before or after correction.

## 5 DISCUSSION

In this study, we have expanded on existing literature for modelling fixative dynamics. The KI model, which assumes a single brain‐wide diffusion coefficient and models the effect of geometry on fixative flux, is closely based on the work by Dawe et al. (2009). We introduced the KT model, which expands on the KI model by incorporating the effects of diffusion anisotropy and tissue specific diffusion coefficients, both provided from voxel‐wise diffusion tensor estimates. When incorporating more realistic models of fixative dynamics (KI and KT), correcting for the effect of fixative concentration was found to yield T_2_ maps with improved homogeneity compared with a simple distance to surface model. The greatest increase in tissue‐type T_2_ homogeneity was achieved using the correction based on the KT model.

Brains fixed with 10% NBF were found to have a strong dependence on predicted concentration maps of fixative outflux. However, brains fixed with 10% formalin were not found to have such a dependence, indicating that the choice of fixative is a primary factor in determining the importance of performing a correction. The distinction in the performance of the correction between the two fixative types implies that the fixative outflux is different for brains fixed with 10% NBF and 10% formalin, surprising given that the only difference between the two fixatives should be the buffer solution. Notably, tissue fixed with NBF has been shown to have superior preservation of tissue microstructure compared with unbuffered formalin (Thavarajah et al., 2012). This suggests that if we are sensitive to a change in fixative concentration due to outflux at the brain surface, the composition of the fixative solution may lead to a more complicated relationship with the estimated T_2_. However, as no external validation was performed of the fixative outflux over the course of this experiment, this hypothesis cannot be tested further.

Brains fixed with 10% NBF and 10% formalin were found to be characterised by very different T_2_ properties, with brains fixed with 10% NBF generating higher overall T_2_ estimates in both grey and white matter (Figure 6). This observation highlights that the choice of fixative has a considerable influence on T_2_ (even when considering the same formalin concentration). Previous work has observed that even the vendor‐specific composition of the fixative solution is a substantial contributor to the estimated MR relaxation properties (Birkl et al., 2018).

T_2_ maps from brains fixed with 10% NBF were found to have a higher correlation with the predicted fixative concentration. Provided such a correction is made, the use of NBF provides benefits for the quality of imaging data. Increased values of T_2_ for brains fixed with 10% NBF versus 10% formalin (Figure 6) provide datasets with higher SNR. Combined with reports of improved structural integrity and immunohistochemical staining for tissue fixed with NBF (Howat & Wilson, 2014; Thavarajah et al., 2012), we recommend that these benefits outweigh the potential confound induced by fixative concentration.

Comparisons with histology reveal that brains fixed with 10% NBF demonstrate an overall stronger correlation with both PLP and ferritin compared with brains fixed with 10% formalin (Figures 10 and 11). For the PLP analysis, brains fixed with 10% NBF and 10% formalin both demonstrate a positive correlation with *R*_2_ (Figure 10), consistent with the observation that myelin is characterised by a short T_2_ (Heath, Hurley, Johansen‐Berg, & Sampaio‐Baptista, 2018; Mackay et al., 1994). The correlation appears to be predominantly driven by established regional differences, with the ACC characterised by the lowest PLP SAFs compared with V2 and M1 (in agreement with reported regional differences in myelination; Glasser et al., 2016; Nieuwenhuys & Broere, 2017).

Correction with the KT model increased the similarity between the relationships of *R*_2_ with PLP for the two fixative types. By correcting for the concentration of fixative with the KT model, we reduce the variance of *R*_2_ across different regions of the brain within individual subjects. This correction led to a small decrease in the correlation with PLP for brains fixed with 10% NBF, suggesting that the confound of fixative concentration is artificially inflating the correlation between *R*_2_ and PLP in these brains. Although we would typically expect the removal of confounds to increase correlations, in this case the correlation between *R*_2_ and PLP is predominantly driven by regional differences, which would also be expected to have consistent patterns of fixative concentration. A small (but significant) positive correlation was found between the expected fixative concentration (as simulated by the KT model) and PLP SAF (Figure S6). This suggests that if is there is outflux of fixative at the tissue surface (which leads to a characteristic change in T_2_), that the correlations across different brain regions are partially driven by the fixative concentration. No significant correlation between concentration and PLP SAF was found for brains fixed with 10% formalin (Figure S6), where a small increase in correlation was observed after correction. However, we would expect that correcting for fixative concentration would lead to an improved correlation between *R*_2_ and PLP when differences are not predominantly driven by different brain regions.

Similarly, a small decrease in correlation between *R*_2_ and ferritin SAF was found for brains fixed with 10% NBF after KT correction, with no notable correlation for brains fixed with 10% formalin. Here, no significant correlation was found between fixative concentration and ferritin SAF for either fixative type (Figure S7). Although ferritin is a non‐quantitative estimate of tissue iron store, we would expect an increased ferritin content to correspond to an increase in tissue *R*_2_ for brains fixed with either 10% NBF or 10% formalin (Vymazal et al., 1992). However, there are a number of limitations to our ferritin analysis that could explain this low level of correlation for 10% formalin brains, most notably that ferritin staining quality is highly variable between batches. Although some effort was taken to normalise the results and combine across batches, when combined with the limited number of regions where ferritin staining data is available makes us particularly sensitive to outliers. Further details of these limitations have been described in detail in a recent publication from our group (Wang et al., 2020). We are currently exploring alternative approaches to more accurately quantify the ferritin content of tissue (Kor et al., 2021). In addition, we are aiming to move away from simple summary measurements across ROIs when performing cross‐scale comparisons between histology & MRI, most notably with the development of a toolbox to directly coregister histology slides to MRI images (Huszar et al., 2019). This will enable us to perform more sophisticated voxelwise comparisons between the MRI and histology data.

In this study, no external validation of the outflux of fixative from the post‐mortem brains was performed. Therefore, while we observe a correlation between our concentration distribution and the T_2_ estimates in brains fixed with 10% NBF, we cannot confirm that this is due to fixative outflux. Although correction with the KT model does appear to remove inhomogeneity in these brains (e.g., Figure 9 and Table 1), the inconsistencies between the two fixative types remain unexplained and requires further exploration. One approach would be to perform T_2_ mapping at multiple time intervals in a single post‐mortem brain, using an experimental design similar to previous work modelling the influx of fixative in tissue (Dawe et al., 2009; Yong‐Hing, Obenaus, Stryker, Tong, & Sarty, 2005). By correlating the changes in T_2_ with the estimated fixative concentration from our simulations, we would be able to validate the proposed fixative outflow, and obtain a more robust estimate of β without the influence of different tissue types and biologically meaningful variations in T_2_. A second approach would be to perform an experiment placing a post‐mortem brain in a scanning medium with different concentrations of fluorinert and formalin. By correlating the measured change in T_2_ with simulations of fixative dynamics in different external mediums, we could similarly validate the observation and obtain a robust estimate of β.

The estimate of β obtained in white matter lead to improved homogeneity in grey matter, in agreement with the expectation that the relationship between T_2_ and fixative concentration is a diffusion driven process which does not depend on other tissue‐specific properties. However, the increase in homogeneity in grey matter is reduced in comparison to the increase in white matter (Figure 7). As our fitting approach does not disentangle between biologically meaningful changes in T_2_ and changes due to fixative outflux, it is reasonable to expect that some biological variation could influence the estimate of β, reducing the translation to other tissue types. Alternatively, there may be more sophisticated mechanisms that drive the relationship between T_2_ and fixative concentration which have not been identified here.

**FIGURE 12:**
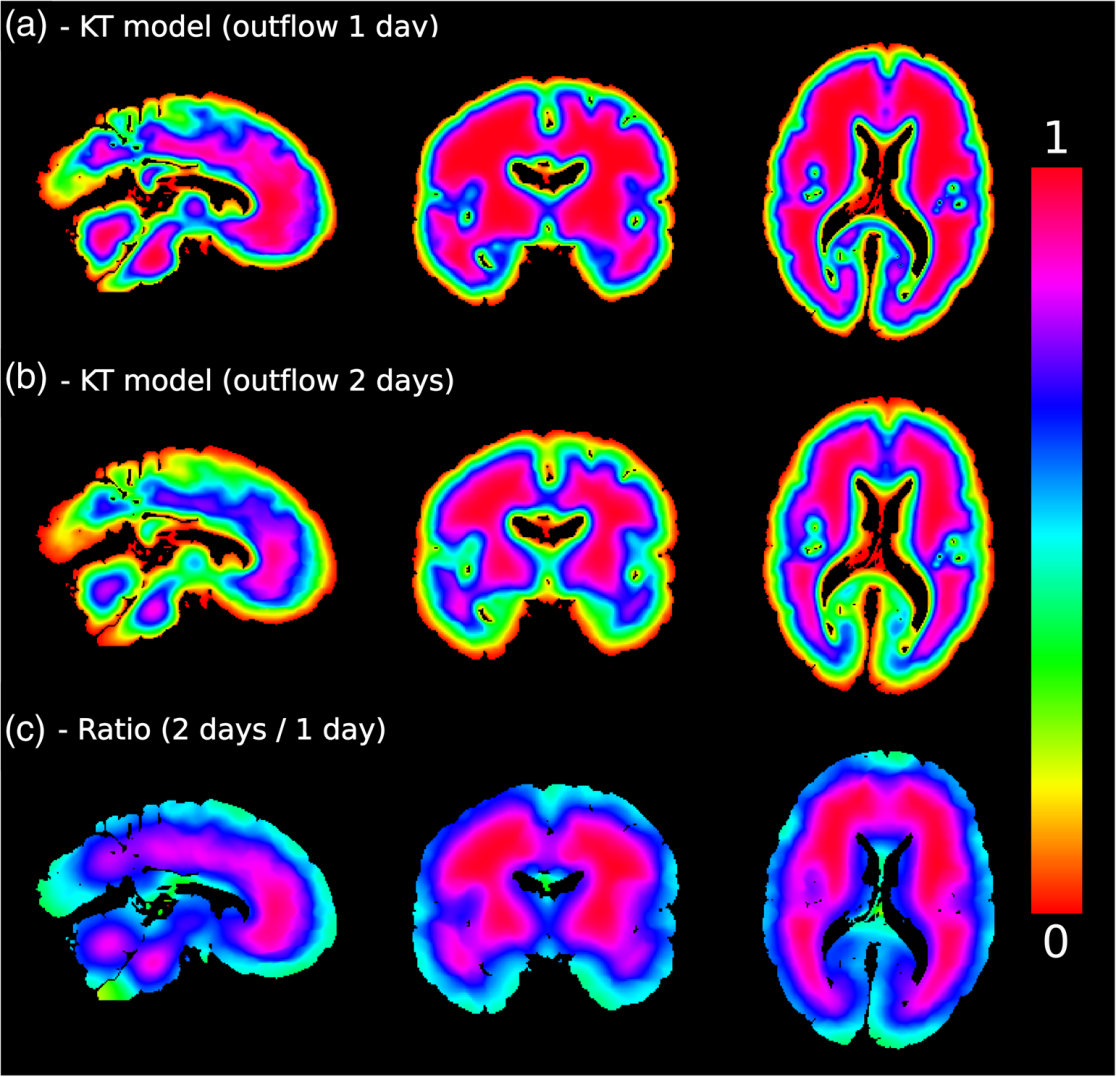
Non‐linearity of the KT model. Here we display simulations of outflux for one (a) and two (b) days using the KT model. The concentration distribution across the brain does not scale linearly with time. This leads to a ratio map (c) that does not reflect the same value across the entire brain. It is therefore recommended to have precise recordings of influx/outflux duration in order to use this approach effectively

There are several further limitations to this study. First, the KI and KT simulations have a strong dependency on the outflux duration. The duration of time between the brains being placed in fluorinert and scanning was not accurately recorded for each individual sample, with 48 hr chosen as an approximate time between these two events. However, our simulations additionally reveal that the concentration distribution does not evolve linearly with time (Figure 12). Precise knowledge of this time period is recommended for accurately simulating the effects of fixative outflux. Similarly, the choice of b‐value in the diffusion MRI experiment may lead to different diffusivity estimates in the post‐mortem tissue sample (due to non‐Gaussian diffusion within tissue; De Santis, Gabrielli, Palombo, Maraviglia, & Capuani, 2011) and thus differences in the concentration profile. Second, the TSE sequence used in this study was highly sensitive to B_1_, requiring the use of an EPG fitting approach to estimate our T_2_ maps (detailed in Supporting information  S1). We additionally investigated whether any of the observed correlations could be attributed to the B_1_ distribution, which has a broadly similar spatial profile to the outflux models used in this study. Although regressing out the B_1_ distribution did lead to a decrease in inhomogeneity (Figure S8 and Table S3), this decrease in inhomogeneity was lower in comparison to the D2S, KI and KT model over brains fixed with 10% NBF, and similar in performance for brains fixed with 10% formalin where the concentration correction did not lead to any significant change in inhomogeneity.

Application of the KT model in this study used diffusion tensor estimates acquired in the same post‐mortem brain to model fixative dynamics. However, for post‐mortem studies that do not include diffusion MRI as part of their acquisition, the use of a diffusion‐tensor atlas (e.g., the HCP0165 standard space DTI template; Jenkinson, Beckmann, Behrens, Woolrich, & Smith, 2012) could be explored as an alternative approach. The KI model (which additionally demonstrated improved performance compared with the phenomenological D2S model) provides a simple method to model fixative dynamics if no diffusion measures are available in a given brain, requiring only a tissue mask and a single estimated diffusion coefficient to simulate. The KT model incorporates the full information provided with diffusion tensor measurements, both in terms of voxel‐wise tissue diffusivities and diffusion anisotropy. Overall, comparisons between the KI and KT model performed in this manuscript can be used to inform investigators of the benefits of incorporating diffusion MRI data when simulating fixative dynamics, building on previous work (Dawe et al., 2009).

There are two key differences between the KI and KT model presented in this manuscript: the incorporation of 1. voxelwise diffusion coefficients and 2. diffusion anisotropy. To investigate if one of these components alone can explain the differences between the KI and KT model, we simulated the KI model incorporating the voxelwise mean diffusivity maps, as opposed to a global diffusivity scalar. We found that this model yielded very similar results to the KT model, as shown in Figure S9 and Table S4. This suggests that the predominant driver of the KT model is the incorporation of voxelwise diffusivities, highlighting the benefits afforded by incorporating realistic diffusion estimates over the KI model. However, given that voxelwise diffusivity estimates are often accompanied by diffusion anisotropy measures, our analysis does not demonstrate any advantage over the KT model.

A further question arises as to the choice of diffusion coefficient used for the KI model simulations, which in this manuscript was set to the mean diffusivity per brain (average *D* = 2.4 ± 1.5 × 10^−4^ mm^2^/s across the brains). To investigate the influence this choice has on results, we repeated the KI model simulations twice with two different diffusion coefficients, set to the average in white matter (*D* = 1.37 ± 0.39 × 10^−4^ mm^2^/s) or grey matter (*D* = 3.1 ± 1.5 × 10^−4^ mm^2^/s) in each brain. We found that the relationships between T_2_ and concentration (Figure S10) were consistent with the KI and KT model (Figures 7 and 8), demonstrating an improved homogeneity over grey and white matter for brains fixed with 10% NBF, but no change for 10% formalin. Correction with the white matter diffusivity led to an improved homogeneity for grey matter for NBF brains, but a considerable reduction in white matter homogeneity versus the original KI model (confirmed in Table S5). Correction with the grey matter diffusivity lead to a slight reduction in homogeneity over grey matter versus the original KI model for NBF brains. Neither approach demonstrated improved performance over the KT model. Taken together, this suggests that the choice of average mean diffusivity over the entire brain is a good compromise for simulating the KI model. However, improved performance can still be found for incorporating the mean diffusivity maps (Figure S9 and Table S4), or better still with the full KT model.

This work forms part of a larger project (Pallebage‐Gamarallage et al., 2018) investigating the pathology of ALS through the combination of post‐mortem MRI and immunohistochemical staining within the same tissue sample, to determine how changes in tissue composition gives rise to measured changes in our MR signal. In order to accurately map these relationships, it is essential to remove any potential confounds which could mask out subtle changes in the MR signal due to tissue pathology, or drive spurious relationships in our data. In this manuscript, we focused on using the KT model to correct for fixative concentration due to the outflux of fixative. However, it would be possible to extend this approach to other challenges in post‐mortem imaging. One example is the estimation of a voxelwise post‐mortem delay. When a brain is fixed, fixative penetrates slowly into brain tissue (Figure 2). By modelling the influx of fixative into tissue, it would be possible to generate a voxelwise estimate of the time required for any individual voxel to become fully fixed. This could additionally be modelled and removed as a confound in the data. A voxel‐wise post‐mortem delay (Shepherd et al., 2009b) might be predictive of effects related to cross‐linking of tissue, which in turn may be reflected in MR‐relevant properties like T_1_.

## 6 CONCLUSION

We introduced the KT model of fixative dynamics in tissue, which incorporates diffusion anisotropy and tissue‐specific diffusion properties. Based on this model, we demonstrated that the resulting concentration map can be used to remove confounds from MR images. T_2_ maps acquired in whole post‐mortem brains reveal a spatial profile consistent with a model of fixative outflux in brains fixed with 10% NBF, with the KT model yielding the greatest reduction in inhomogeneity in T_2_ across both grey and white matter. Results were found to be strongly dependent on the type of fixative, with further exploration required to verify whether the observed changes can be attributed to fixative outflux, and the contribution of the buffer solution to this process.

## CONFLICT OF INTEREST

The authors declare no conflicts of interest.

## AUTHOR CONTRIBUTIONS

**Benjamin C. Tendler:** Established the diffusion MRI processing pipeline, contributed to the development of the T_2_ mapping processing pipeline & KI/KT modelling framework, processed the MRI data, performed data analysis and wrote the manuscript. **Feng Qi:** Established the T_2_ mapping pipeline & KI/KT modelling framework, and contributed to the data analysis. **Sean Foxley:** Established the post‐mortem MRI protocols and performed data acquisition. **Menuka Pallebage‐Gamarallage:** Carried out systematic sampling, block face photography, immunohistochemistry and histology analysis. **Ricarda A.L. Menke:** Generated ROI masks. **Olaf Ansorge:** Developed the sampling strategy and advised on histology analysis. **Samuel A. Hurley:** Conceived the study design, contributed to the development of the T_2_ mapping protocols, KI/KT modelling framework and data analysis. **Karla L. Miller:** Conceived the study design, contributed to the development of the T_2_ mapping protocols, KI/KT modelling framework, data analysis and the establishment of the post‐mortem MRI protocol. All authors read and edited the manuscript.

## ETHICS STATEMENT

The study was conducted under the Oxford Brain Bank's generic Research Ethics Committee approval (15/SC/0639).

## Supporting information


**Table S1** Characteristics of each brain used in this study. ALS, amyotrophic lateral sclerosis; FTD, frontotemporal dementia; NBF, neutral buffered formalin.
**Table S2** Acquisition parameters for the TSE scans used in this study. Details of individual brains provided in Table S1.
**Figure S1** Motivation for the EPG framework. When the refocusing angle = 180° (a: red line), the TSE signal evolves via a mono‐exponential decay. However, at different flip angles the signal evolution can deviate substantially from a mono‐exponential signal model (a: purple, blue and green lines). At 7 T, brain samples experience B_1_ inhomogeneity, leading to a spatially varying flip angle across the brain (b). Fitting these data with a mono‐exponential model leads to a T_2_ map that depends strongly on the B_1_ profile (c). An EPG framework is able to account for the spatially varying flip angle across the brain, leading to T_2_ maps with more homogeneous contrast (d). (a) simulated using an EPG framework (TE = 10–60 ms with a 10 ms echo spacing, T_2_ = 30 ms), with resulting curves normalised to the signal at TE = 10 ms to aid visualisation of the deviation from a mono‐exponential decay. Note that there is a degeneracy when α > 180°, where flip angle a produces the same signal evolution as 360° – α.
**Figure S2** T_2_ estimates with EPG and smoothing B_1_. Step 1 – Our EPG model estimates B_1_ maps a with decreasing B_1_ as the brain boundary is approached (a), in addition to T_2_ maps that do not have a strong dependence on the B_1_ profile (b). The B_1_ profile is expected to be smoothly varying across the brain. However, anatomical contrast is visible within the B_1_ map (a: blue arrow), in addition to sharp discontinuities in areas close to the brain boundary (a: green arrows), regions associated with low SNR. This leads to artefacts and subtle contrast changes in the resulting T_2_ map (b: blue and green arrows). Step 2 – By smoothing the B_1_ maps with a polynomial filter (c) and fixing B_1_ to the resulting map in a second stage estimate of T_2_, we obtain consistent T_2_ estimates across the brain (d). Here the B_1_ maps (a and c) are displayed between 0 and 1, with the T_2_ maps (b and d) displayed between 0 and 60 ms. N.B (d) does not include the regularisation step [Equation (S3)].
**Figure S3** Addition of regularisation for our T_2_ estimates. In areas of low B_1_ in close proximity to the brain boundary, the TSE data had very low SNR. In some of our T_2_ maps, this lead to spurious T_2_ estimates [visible in (a)]. The addition of regularisation (b) via fitting with Equation (S3) bought the T_2_ values within these regions into agreement with the surrounding tissue. Within other areas of tissue, the regularisation led to negligible changes in T_2_ (c).
**Figure S4** Artefacts arising in fixative concentration simulations due to spuriously high diffusion coefficients. (a) displays an axial slice of the principal eigenvalue (L1) map of a single post‐mortem brain, with (b) displaying a mask of voxels containing the spuriously high diffusion coefficients (>1 × 10^–3^ mm^2^/s). (c,d) display the concentration distribution map after 2 days of fixative outflow as described in the Methods (Main Text) without (c) and with (d) correction for high diffusion coefficients. Failure to perform the correction leads to artefacts in the resulting concentration map (c), most apparent in the regions highlighted by the orange arrows. Correction of these voxels (d) results in a smooth concentration map. Mean diffusivity map (a) displayed between 0 and 5 × 10^–3^ mm^2^/s, concentration distributions (c,d) displayed between 0 and 1.
**Figure S5** Distribution of white / grey matter. For all three models, white and grey matter are not evenly distributed with respect to distance from the nearest surface or fixative concentration. Plots generated over all 14 brains, with error bars representing the SD between brains. Voxels <2 mm from the nearest surface were not included in our analysis and are therefore not displayed here.
**Figure S6** Fixative concentration versus PLP SAF across M1, ACC and V2. For brains fixed with 10% NBF, a small (but significant) correlation was found between the concentration of fixative and the PLP SAF, where there is a notable distinctions in the fixative concentration and PLP SAF in individual regions (e.g., ACC and M1: hand region). For Brains fixed with 10% formalin, no such relationship was observed.
**Figure S7** Fixative concentration versus ferritin SAF across M1, ACC and V2. No significant correlation was observed between fixative concentration and ferritin SAF for brains fixed with 10% NBF or 10% formalin. Note that as the ferritin SAFs were normalised for the two batches, the SAF values can be positive and negative and are not restricted to a range between 0 and 1. As the ACC and V2 regions were included in both batches, the ferritin SAFs were averaged prior to analysis and plotting.
**Figure S8** T_2_ versus B_1_ over white and grey matter for all post‐mortem brains fixed with 10% NBF and 10% formalin. The corrections presented in this work assume that presence of fixative in tissue is the source of variation in T_2_ across the brains. However, the predicted patterns of fixative are similar to the spatial pattern of B_1_. Here, we consider how much of the variation in T_2_ may be explained by the B_1_ spatial profile. A dependency is observed versus B_1_ in our post‐mortem cohort (a), with a stronger effect in brains fixed with 10% NBF (left) versus 10% formalin (right), consistent with the other three models investigated in this study (Main Text Figures 7 and 8). However, regressing out the influence of B_1_ using Equation (6) (b) leads to a higher remaining inhomogeneity as shown in Table S3 versus the D2S, KI and KT models for the brains fixed with 10% NBF (Main Text Table 1), and similar performance (no change) for brains fixed with 10% formalin. Results displayed as the mean ± standard deviation across brains.
**Table S3** Inhomogeneity over white and grey matter for brains fixed with 10% NBF and 10% formalin – correction with B_1_. Correction with B_1_ gave rise to a reduction in inhomogeneity across both grey and white matter for brains fixed with 10% NBF. However, this reduction in inhomogeneity did not reach significance, and was a smaller improvement versus the D2S, KI and KT models (Main Text Table 1). Little change was found in brains fixed with 10% formalin, consistent with the corrections using the D2S, KI and KT models (Main Text Table 1). The *p*‐values comparing the change in inhomogeneity for the B_1_ correction displayed in brackets.
**Figure S9** T_2_ versus concentration for the KI model incorporating mean diffusivity maps. The KT model presented in this work incorporates both diffusion anisotropy and voxel‐specific diffusion coefficients to simulate fixative dynamics. Here, we consider how much of the variation in T_2_ can be explained by the voxel‐specific diffusion coefficients alone. Simulations using the KI model incorporating voxel‐wise mean diffusivity maps yields very similar T_2_‐concentration distributions to the KT model (Main Text Figures 7 and 8). Regressing out the influence of the KI model incorporating these mean diffusivity maps using Equation (6) (b) leads to a marginally higher remaining inhomogeneity (Table S4) compared with the KT model for brains fixed with 10% NBF (Main Text Table 1), and similar performance (no change) for brains fixed with 10% formalin. This indicates that voxel‐wise variations in diffusivity are the predominant driver of fixative dynamics in the KT model, with diffusion anisotropy less crucial for reducing T_2_ inhomogeneity. Results displayed as the mean ± *SD* across brains.
**Table S4** Inhomogeneity over white and grey matter for brains fixed with 10% NBF and 10% formalin – correction with the KI model incorporating mean diffusivity maps. Correction with the KI model incorporating mean diffusivity maps led to inhomogeneity estimates similar to the KT model for brains fixed with both 10% NBF and 10% formalin (Main Text Table 1). The *p*‐values comparing the change in inhomogeneity for the model correction displayed in brackets.
**Figure S10** T_2_ versus concentration for the KI model incorporating white and grey matter diffusivity estimates. The KI model presented in this work simulates fixative dynamics utilising a single, global scalar of diffusivity, set as the average mean diffusivity over the entire brain. Here we repeat the simulations setting the global scalar as the average mean diffusivity over (a) white matter and (b) grey matter. Regressing out the influence of the KI model using Equation (6) with the average over white matter (a) leads to an improved homogeneity for grey matter for NBF brains, but a considerable reduction in white matter homogeneity versus the original KI model (confirmed in Table S5). Correction with the average over grey matter for NBF brains (b) leads to a slight reduction in homogeneity over grey matter versus the original KI model. Similar performance (no change) was found for brains fixed with 10% formalin. Neither model demonstrated improved performance over the KT model. Results displayed as the mean ± standard deviation across brains.
**Table S5** Inhomogeneity over white and grey matter for brains fixed with 10% NBF and 10% formalin – correction with the KI model incorporating white and grey matter diffusivity estimates. Correction with the KI model incorporating the average mean diffusivity over white matter (middle column) and grey matter (right column). Similar performance was found for brains fixed with 10% formalin versus the KI and KT model (Main Text Table 1). However, the white matter diffusivity leads to an improved homogeneity for grey matter for NBF brains, but a considerable reduction in white matter homogeneity versus the original KI model. Correction with the average over grey matter for NBF brains leads to a slight reduction in homogeneity over grey matter versus the original KI model. Neither model demonstrated improved performance over the KT model. The *p*‐values comparing the change in inhomogeneity for the model correction displayed in brackets.

## Data Availability

Code associated with manuscript is available at https://github.com/BenjaminTendler/KT_model. Imaging datasets will be made available on the Digital Brain Bank (https://open.win.ox.ac.uk/DigitalBrainBank/).

## APPENDIX A.

Equation (1) is discretised over space and time to obtain (Jbabdi et al., 2005):

(A1)
$$\begin{array}{l} \frac{C^{n+1}\left(X,Y,Z\right)-C^{n}\left(X,Y,Z\right)}{\tau }=D_{11}\left(X,Y,Z\right)\cdot \frac{C^{n}\left(X+1,Y,Z\right)+C^{n}\left(X-1,Y,Z\right)-2\cdot C^{n}\left(X,Y,Z\right)}{\Delta X^{2}}\\ \;+D_{22}\left(X,Y,Z\right)\cdot \frac{C^{n}\left(X,Y+1,Z\right)+C^{n}\left(X,Y-1,Z\right)-2\cdot C^{n}\left(X,Y,Z\right)}{\Delta Y^{2}}\\ \;+D_{33}\left(X,Y,Z\right)\cdot \frac{C^{n}\left(X,Y,Z+1\right)+C^{n}\left(X,Y,Z-1\right)-2\cdot C^{n}\left(X,Y,Z\right)}{\Delta Z^{2}}\\ \;+D_{12}\left(X,Y,Z\right)\cdot \frac{C^{n}\left(X+1,Y+1,Z\right)+C^{n}\left(X-1,Y-1,Z\right)-C^{n}\left(X+1,Y-1,Z\right)-C^{n}\left(X-1,Y+1,Z\right)}{4\cdot \text{ΔXΔY}}\\ \;+D_{13}\left(X,Y,Z\right)\cdot \frac{C^{n}\left(X+1,Y,Z+1\right)+C^{n}\left(X-1,Y,Z-1\right)-C^{n}\left(X+1,Y,Z-1\right)-C^{n}\left(X-1,Y,Z+1\right)}{4\cdot \text{ΔXΔZ}}\\ \;+D_{23}\left(X,Y,Z\right)\cdot \frac{C^{n}\left(X,Y+1,Z+1\right)+C^{n}\left(X,Y-1,Z-1\right)-C^{n}\left(X,Y+1,Z-1\right)-C^{n}\left(X,Y-1,Z+1\right)}{4\cdot \text{ΔYΔZ}}\\ \;+{\tilde{D}}_{1}\left(X,Y,Z\right)\cdot \frac{C^{n}\left(X+1,Y,Z\right)-C^{n}\left(X-1,Y,Z\right)}{2\cdot \mathrm{\Delta X}}+{\tilde{D}}_{2}\left(X,Y,Z\right)\cdot \frac{C^{n}\left(X,Y+1,Z\right)-C^{n}\left(X,Y‐1,Z\right)}{2\cdot \Delta Y}\\ \;+{\tilde{D}}_{3}\left(X,Y,Z\right)\cdot \frac{C^{n}\left(X,Y,Z+1\right)-C^{n}\left(X,Y,Z-1\right)}{2\cdot \mathrm{\Delta Z}} \end{array}\;\;$$

where, $C^{n}\left(X,Y,Z\right)$ is the concentration of fixative at iteration n in voxel $\left(X,Y,Z\right)$; ΔX/ΔY/ΔZ is the voxel dimension along each axis; τ is the time step (per iteration), $D_{\mathrm{ij}}\left(X,Y,Z\right)$ is the component ij of diffusion tensor $D\left(X,Y,Z\right)$, ${\tilde{D}}_{i}\left(X,Y,Z\right)=\frac{D_{i1}\left(X+1,Y,Z\right)-D_{i1}\left(X-1,Y,Z\right)}{2\cdot \Delta X}+\frac{D_{i2}\left(X,Y+1,Z\right)-D_{i2}\left(X,Y-1,Z\right)}{2\cdot \Delta Y}+\frac{D_{i3}\left(X,Y,Z+1\right)-D_{i3}\left(X,Y,Z-1\right)}{2\cdot \Delta Z}$. By rearranging Equation (A1), the spatial distribution of fixative concentration at iteration n+1 ($C^{n+1}$) can be estimated from $C^{n}$ and D.
