# Supplementary material for: A method to remove the influence of fixative concentration on postmortem T2 maps using a kinetic tensor model
Source: Hum Brain Mapp. 2021 Sep 20;42(18):5956–72. doi: 10.1002/hbm.25661 (PMC8596944; doi:10.1002/hbm.25661)
Supplement: Supplementary file 1 — Table S1 Characteristics of each brain used in this study. ALS, amyotrophic lateral sclerosis; FTD, frontotemporal dementia; NBF, neutral buffered formalin. Table S2 Acquisition parameters for the TSE scans used in this study. Details of individual brains provided in Table S1. Figure S1 Motivation for the EPG framework. When the refocusing angle = 180° (a: red line), the TSE signal evolves via a mono‐exponential decay. However, at different flip angles the signal evolution can deviate substantially from a mono‐exponential signal model (a: purple, blue and green lines). At 7 T, brain samples experience B1 inhomogeneity, leading to a spatially varying flip angle across the brain (b). Fitting these data with a mono‐exponential model leads to a T2 map that depends strongly on the B1 profile (c). An EPG framework is able to account for the spatially varying flip angle across the brain, leading to T2 maps with more homogeneous contrast (d). (a) simulated using an EPG framework (TE = 10–60 ms with a 10 ms echo spacing, T2 = 30 ms), with resulting curves normalised to the signal at TE = 10 ms to aid visualisation of the deviation from a mono‐exponential decay. Note that there is a degeneracy when α > 180°, where flip angle a produces the same signal evolution as 360° – α. Figure S2 T2 estimates with EPG and smoothing B1. Step 1 – Our EPG model estimates B1 maps a with decreasing B1 as the brain boundary is approached (a), in addition to T2 maps that do not have a strong dependence on the B1 profile (b). The B1 profile is expected to be smoothly varying across the brain. However, anatomical contrast is visible within the B1 map (a: blue arrow), in addition to sharp discontinuities in areas close to the brain boundary (a: green arrows), regions associated with low SNR. This leads to artefacts and subtle contrast changes in the resulting T2 map (b: blue and green arrows). Step 2 – By smoothing the B1 maps with a polynomial filter (c) and fixing B1 to the resulting map in a sec [file HBM-42-5956-s001.pdf]

Supporting Information

Brain	Clinical diagnosis	Post-mortem delay (days)	Fixative type	Time in fixative before scanning (days)
**1**	Control	3	10% NBF	115
**2**	Control	3	10% NBF	45
**3**	Control	3	10% NBF	48
**4**	ALS	1	10% NBF	35
**5**	ALS	2	10% NBF	87
**6**	ALS	3	10% NBF	94
**7**	ALS	2	10% NBF	139
**8**	ALS	4	10% Formalin	178
**9**	ALS	3	10% Formalin	114
**10**	ALS	7	10% Formalin	137
**11**	ALS	4	10% Formalin	94
**12**	ALS	3	10% Formalin	158
**13**	ALS	Data Unavailable	10% Formalin	96
**14**	ALS + FTD	2	10% Formalin	283

Table S1: **Characteristics of each brain used in this study**. Here ALS – Amyotrophic Lateral Sclerosis, FTD – Frontotemporal Dementia, NBF – Neutral Buffered Formalin.

Brain	1-3, 7	8	9-12	4,13	5-6,14
**No. echoes**	6	6	6	6	6
**TE (ms)**	13, 25, 38, 50, 63, 76	11, 23, 34, 46, 57, 69	11, 23, 34, 46, 57, 69	11, 23, 34, 46, 57, 69	10, 20, 30, 40, 50, 60
**TR (ms)**	1000	1000	1000	1000	1000
**Resolution (mm^3)**	0.9 x 0.9 x 0.9	0.8 x 0.8 x 1.6	0.8 x 0.8 x 1.6	0.65 x 0.65 x 1.3	1.0 x 1.0 x 1.2
**Bandwidth (Hz/pixel)**	166	195	195	163	199
**Turbo factor**	6	6	6	6	6
**Time per TE (minutes)**	36	26	16	18	24
**Slice resolution (%)**	100	100	50	50	100

Table S2: **Acquisition parameters for the TSE scans used in this study**. Details of individual brains provided in Table S1.

Estimating T_2 using an Extended Phase Graph (EPG) model

The TSE sequence is characterised by a train of RF pulses (90°-180°-180°-180°-…), where the signal is sampled after each 180° pulse. Assuming a single tissue compartment and a perfect 180° pulse, the TSE signal evolves via a characteristic mono-exponential decay (Fig. S1a – red line). At 7T, B_1 inhomogeneity produces a spatially varying flip angle across the brain (Fig. S1b), leading to refocusing pulses that vary from 180°. Under these conditions, the signal evolution can deviate substantially from a mono-exponential signal model (Fig. S1a – purple, blue and green lines). Fitting a mono-exponential signal model to these data will lead to estimates of T_2 that strongly depend on the B_1 profile (Fig. S1c).

Extended Phase Graphs (EPG) provide a more accurate description of signal evolution under a variety of MRI sequences and conditions (1–3). In this work, we use an EPG framework to describe the signal evolution of the TSE sequence under a refocusing pulse of flip angle α. Our approach fits the measured TSE signal to estimate the voxelwise T_2 and α (via estimation of B_1). Fitting with an EPG model is shown to reduce the bias on T_2 estimates in areas of low B_1, leading to more homogeneous T_2 maps across the brain (Fig. S1d).

To achieve this, we performed a two-step fitting approach, where we first fit the TSE signal to an EPG model to estimate T_2 and B_1 across the brain. It was found that in regions of very low B_1, sharp discontinuities were observed in the B_1 maps. Given that B_1 is expected to smoothly vary across the brain, we subsequently smoothed the B_1 map and repeated the fitting for T_2, keeping B_1 as a fixed parameter.

Step one:

For each postmortem brain we simulated the TSE sequence (parameters provided in Table S2) under an EPG framework, obtaining voxelwise estimates of B_1 and T_2 by minimising:

$$\min_{B_1,T_2}\left\|\mathrm{TSE}_{\mathrm{exp}}(x,y,z,\mathrm{TE}_{1:6}) - \mathrm{TSE}_{\mathrm{sim}}(B_1,T_2,T_1,\mathrm{TE}_{1:6})\right\|_2^2, \quad \text{[S1]}$$

where $\mathrm{TSE}_{\mathrm{exp}}$ is the experimental TSE data over all six echoes ($\mathrm{TE}_{1:6}$) and $\mathrm{TSE}_{\mathrm{sim}}$ is the simulated TSE signal using the EPG framework given a value of B_1, T_2, T_1, and the echo times. To avoid fitting for the signal amplitude (S_0), the experimental data and simulated signal were normalised (e.g. division by $\left(\sum_{j=1}^{6}\mathrm{TSE}_{\mathrm{j}}^2\right)^{0.5}$). In a series of evaluations, T_1 was found to have very little effect on our T_2 estimates, after which it was set equal to a fixed

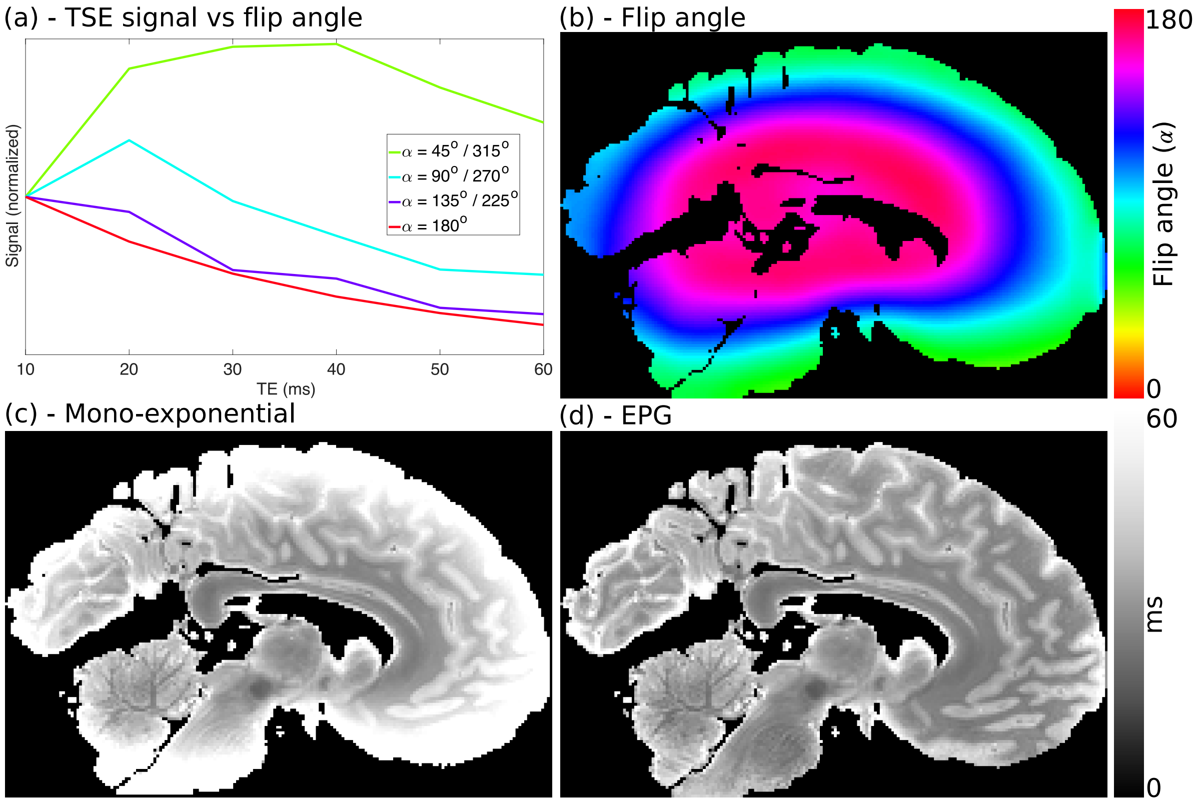

Figure S1: **Motivation for the EPG framework**. When the refocusing angle = 180° (a – red line), the TSE signal evolves via a mono-exponential decay. However, at different flip angles the signal evolution can deviate substantially from a mono-exponential signal model (a – purple, blue and green lines). At 7T, brain samples experience B_1 inhomogeneity, leading to a spatially varying flip angle across the brain (b). Fitting these data with a mono-exponential model leads to a T_2 map that depends strongly on the B_1 profile (c). An EPG framework is able to account for the spatially varying flip angle across the brain, leading to T_2 maps with more homogeneous contrast (d). (a) simulated using an EPG framework (TE = 10 – 60 ms with a 10 ms echo spacing, T_2 = 30 ms), with resulting curves normalised to the signal at TE = 10 ms to aid visualisation of the deviation from a mono-exponential decay. Note that there is a degeneracy when $\alpha > 180°$, where flip angle α produces the same signal evolution as $360° - \alpha$.

constant (450 ms - the approximate value of measured T_1 in our postmortem datasets). Fitting was performed in MATLAB (version 2019b, The MathWorks, Inc., Natick, MA) based on code from (3). Eq. [S1] was minimised using *lsqnonlin*.

Figures S2a and b show the resulting B_1 and T_2 maps for a single postmortem brain fit with this approach. The spatial inhomogeneity across the T_2 map is substantially reduced in comparison to the mono-exponential fit (Fig. S1c). However, B_1 maps are expected to vary smoothly across the brain and Fig. S2a reveals changes in B_1 that depend on the tissue contrast (e.g. blue arrow), in addition to underestimation of B_1 close to the brain boundary

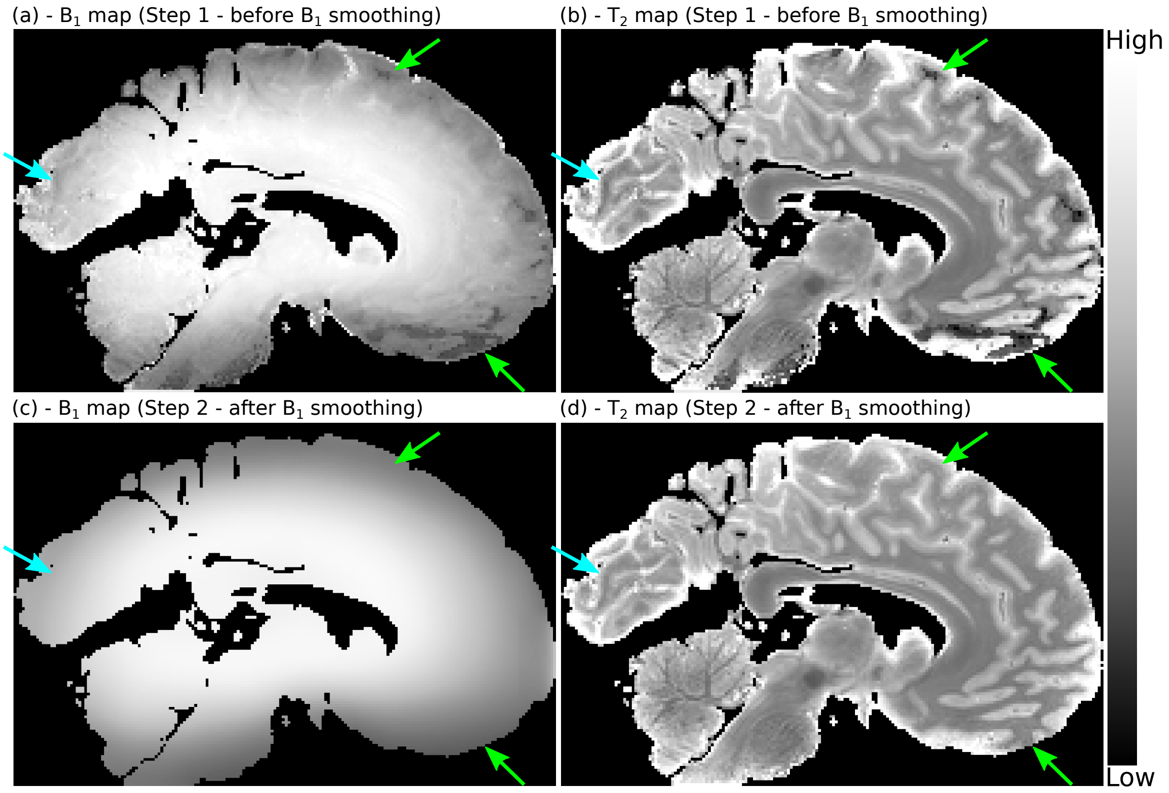

Figure S2: **T_2 estimates with EPG and smoothing B_1.** Step 1 - Our EPG model estimates B_1 maps a with decreasing B_1 as the brain boundary is approached (a), in addition to T_2 maps that do not have a strong dependence on the B_1 profile (b). The B_1 profile is expected to be smoothly varying across the brain. However, anatomical contrast is visible within the B_1 map (a – blue arrow), in addition to sharp discontinuities in areas close to the brain boundary (a - green arrows), regions associated with low SNR. This leads to artefacts and subtle contrast changes in the resulting T_2 map (b – blue and green arrows). Step 2 - By smoothing the B_1 maps with a polynomial filter (c) and fixing B_1 to the resulting map in a second stage estimate of T_2, we obtain consistent T_2 estimates across the brain (d). Here the B_1 maps (a and c) are displayed between 0 and 1, with the T_2 maps (b and d) displayed between 0 and 60 ms. N.B (d) does not include the regularisation step (Eq. [S3]).

(e.g. green arrows). This leads to clear artefacts in the resulting T_2 maps (Fig. S2b), most apparent in regions where B_1 is underestimated (green arrows).

Step two:

To ensure spatial smoothness of B_1, the B_1 maps from step 1 were subsequently filtered using a local 3D polynomial filter (order = 2, kernel volume = 10x10x10 mm^3), with voxels weighted by the inverse of the standard error on the B_1 estimates. Figure S2c displays an example B_1 map after filtering, revealing a smoothly varying B_1 profile across the entire brain.

T_2 estimates were subsequently regenerated across the brain using the smoothed B_1 map as follows:

$$\min_{T_2} \left\| \mathrm{TSE}_{\mathrm{exp}}(x, y, z, \mathrm{TE}_{1:6}) - \mathrm{TSE}_{\mathrm{sim}}(B_1(x, y, z), T_2, T_1, \mathrm{TE}_{1:6}) \right\|_2^2, \qquad \text{[S2]}$$

where B_1 was fixed to the value of the smoothed B_1 map. Figure S2d displays the resulting T_2 map, where the most notable change is in regions of the brain where the B_1 was previously underestimated (Fig. 2d green arrows) – in these regions the T_2 estimates now match those of the surrounding tissue.

In regions very close to the brain boundary (characterised by very low B_1 and low SNR), in some brains the T_2 estimates were found to be highly sensitive to noise, leading to spurious values (Fig. S3a). To correct for this, regularisation was added to Eq. [S2] as follows:

$$\min_{T_2} \left\| \mathrm{W} \cdot [\mathrm{TSE}_{\mathrm{exp}}(x, y, z, \mathrm{TE}_{1:6}) - \mathrm{TSE}_{\mathrm{sim}}(B_1(x, y, z), T_2, T_1, \mathrm{TE}_{1:6})] \right\|_2^2 + \lambda \left\| \mathrm{T}_2 - T_{2,\mathrm{med}} \right\|_2^2, \qquad \text{[S3]}$$

where λ is a regularisation constant ($\lambda = 2$), $T_{2,\mathrm{med}}$ is the median value of T_2 across the entire brain from the first step fitting and W is a scalar weight $\left(\sum_{j=1}^{6} \mathrm{TSE}_{\mathrm{exp,j}}^2\,(x, y, z)\right)^{0.5}$. Figure S3b displays a T_2 map with the addition of regularisation. Differences between the two maps (Fig. S3c) are restricted to these areas of very low B_1 and SNR. Figure 5 (Main Text) displays the T_2 maps fit using this approach for all 14 brains used in our study.

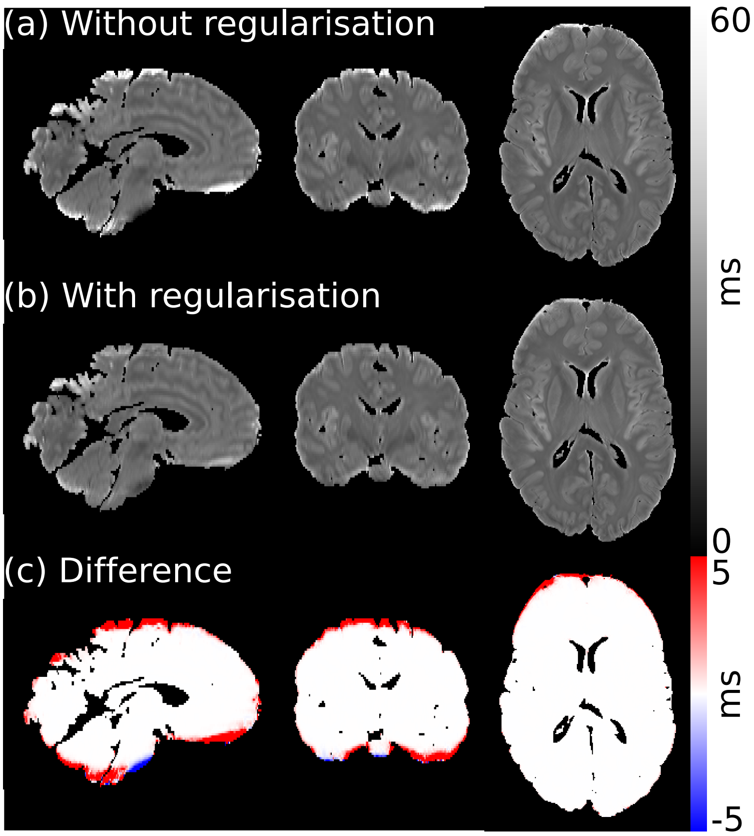

Figure S3: **Addition of regularisation for our T_2 estimates**. In areas of low B_1 in close proximity to the brain boundary, the TSE data had very low SNR. In some of our T_2 maps, this lead to spurious T_2 estimates (visible in (a)). The addition of regularisation (b) via fitting with Eq. [S3] bought the T_2 values within these regions into agreement with the surrounding tissue. Within other areas of tissue, the regularisation led to negligible changes in T_2 (c).

Correcting for spuriously high diffusion coefficients

It is not possible to determine diffusion coefficients in voxels with unreliable signal estimates. In this study, unreliable signal estimates arose (1) in voxels in very close proximity to remaining air bubbles, (2) in regions of extremely low SNR, and (3) due to imperfect masking (resulting in a small number of non-brain tissue voxels remaining in the brain mask, e.g. small quantities of signal-producing formalin remaining in brain sulci and ventricles). In these voxels, diffusion coefficients manifested as spuriously high values on our resulting diffusion tensor estimates.

The number of unreliable voxels was small, with an average of 1.9 ± 2.1% of voxels / brain requiring correction, below 0.6% in 7 of the 14 brains. However, it is critical to correct for these voxels for two reasons. First, an accurate brain shape is important for modelling fixative dynamics - removal of these voxels would lead to a change the brain shape, or holes in the brain. Second, spuriously high diffusion coefficients can lead to artefacts in the resulting fixative concentration maps (Fig. S4). These artefacts arise when a large concentration gradient across neighbouring voxels induces a change in concentration > 1 in a single iteration of the fixative dynamics simulation (Main Text Appendix Eq. [A1]), resulting in negative concentration values. One approach to prevent these artefacts is to reduce the time step per iteration (τ in Main Text Appendix Eq. [A1]). However, this can considerably increase the time required to perform the fixative simulations. Notably, even a single voxel can give rise to this artefact, motivating our use of a threshold to remove the spuriously high diffusion coefficients.

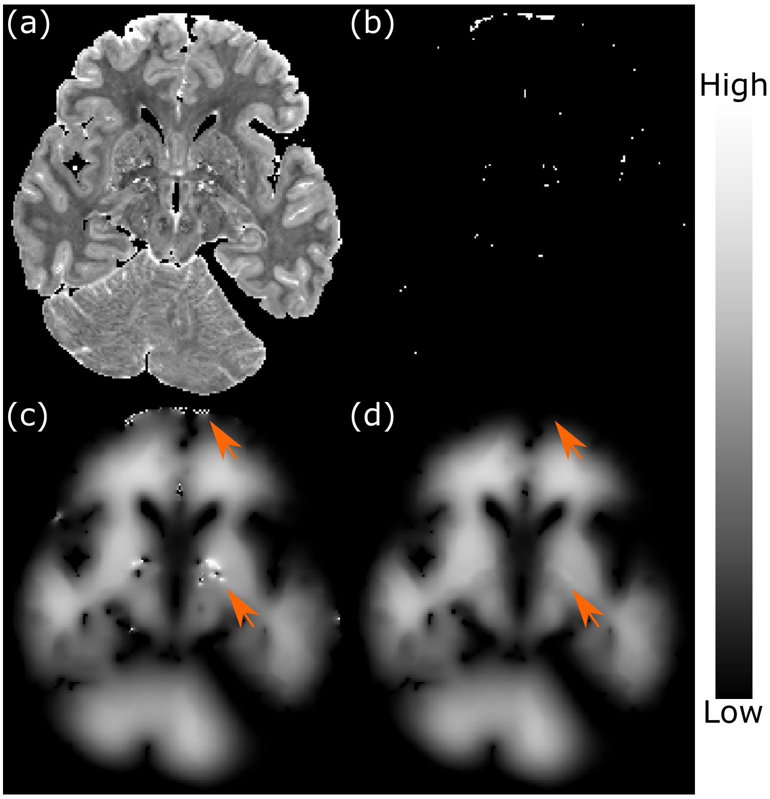

Figure S4: **Artefacts arising in fixative concentration simulations due to spuriously high diffusion coefficients**. (a) displays an axial slice of the principal eigenvalue (L1) map of a single post-mortem brain, with (b) displaying a mask of voxels containing the spuriously high diffusion coefficients (>1 · 10^{-3} mm^2/s). (c) and (d) display the concentration distribution map after two days of fixative outflow as described in the Methods (Main Text) without (c) and with (d) correction for high diffusion coefficients. Failure to perform the correction leads to artefacts in the resulting concentration map (c), most apparent in the regions highlighted by the orange arrows. Correction of these voxels (d) results in a smooth concentration map. Mean diffusivity map (a) displayed between 0 and 5 · 10^{-3} mm^2/s, concentration distributions (c & d) displayed between 0 and 1.

Additional Supporting Figures and Tables

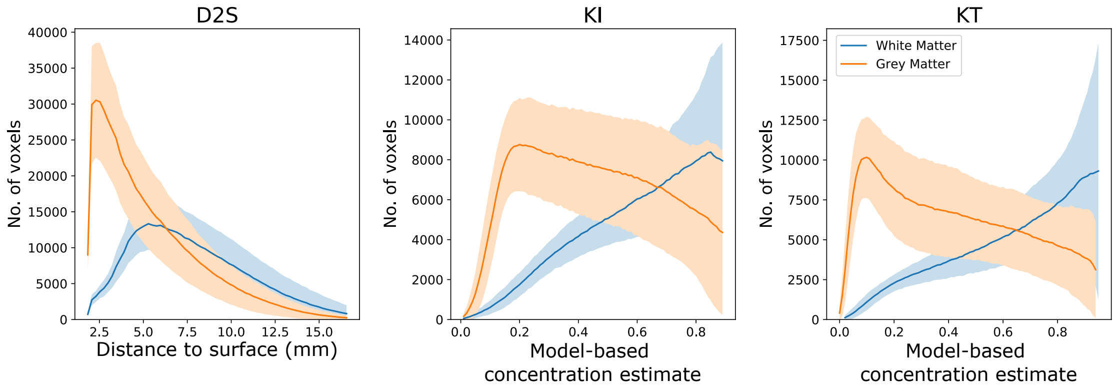

Figure S5: **Distribution of white / grey matter.** For all three models, white and grey matter are not evenly distributed with respect to distance from the nearest surface or fixative concentration. Plots generated over all 14 brains, with error bars representing the standard deviation between brains. Voxels < 2 mm from the nearest surface were not included in our analysis and are therefore not displayed here.

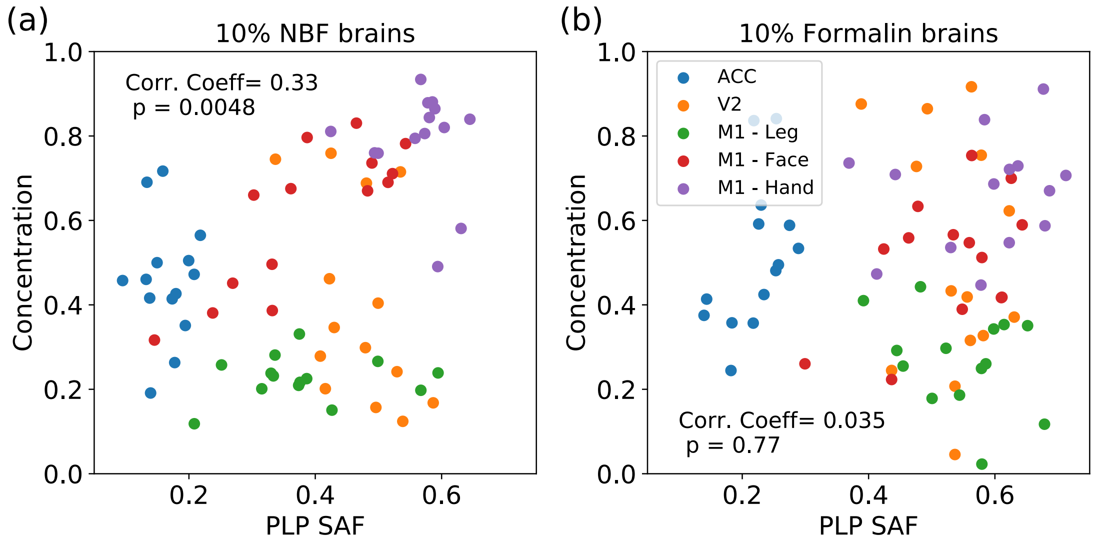

Figure S6: **Fixative concentration vs PLP SAF across M1, ACC and V2.** For brains fixed with 10% NBF, a small (but significant) correlation was found between the concentration of fixative and the PLP SAF, where there is a notable distinctions in the fixative concentration and PLP SAF in individual regions (e.g. ACC and M1 – hand region). For Brains fixed with 10% formalin, no such relationship was observed.

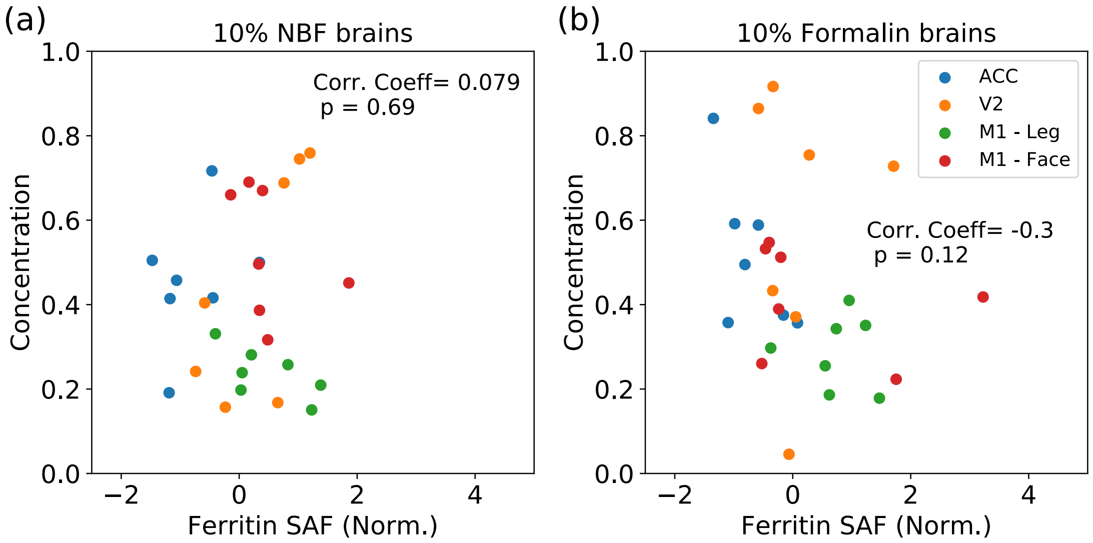

Figure S7: **Fixative concentration vs ferritin SAF across M1, ACC and V2.** No significant correlation was observed between fixative concentration and ferritin SAF for brains fixed with 10% NBF or 10% formalin. Note that as the ferritin SAFs were normalised for the two batches, the SAF values can be positive & negative and are not restricted to a range between 0 and 1. As the ACC and V2 regions were included in both batches, the ferritin SAFs were averaged prior to analysis & plotting.

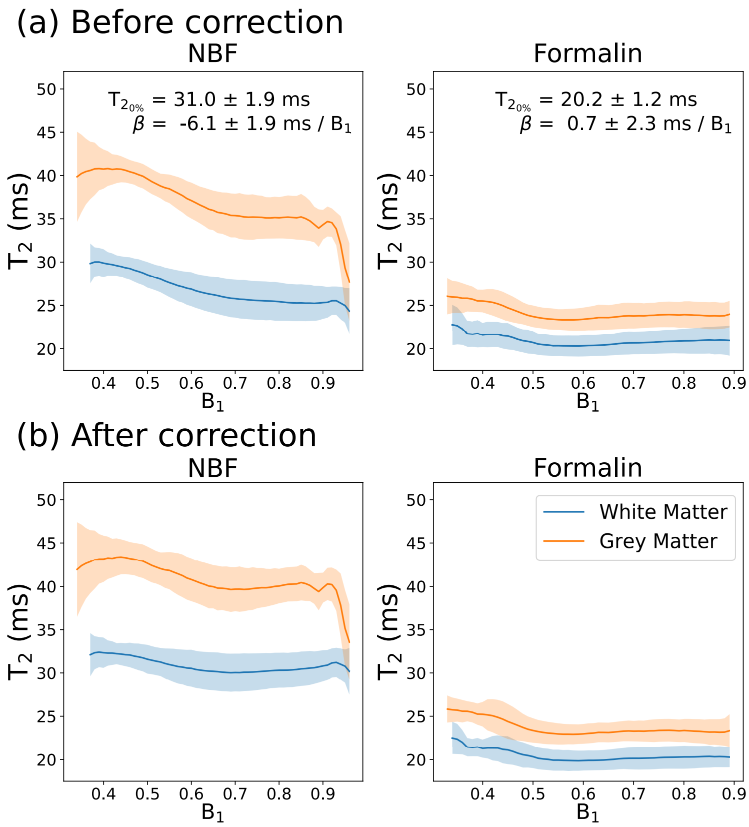

Figure S8: **T_2 vs B_1 over white and grey matter for all post-mortem brains fixed with 10% NBF and 10% formalin.** The corrections presented in this work assume that presence of fixative in tissue is the source of variation in T_2 across the brains. However, the predicted patterns of fixative are similar to the spatial pattern of B_1. Here, we consider how much of the variation in T_2 may be explained by the B_1 spatial profile. A dependency is observed vs B_1 in our post-mortem cohort (a), with a stronger effect in brains fixed with 10% NBF (left) vs 10% formalin (right), consistent with the other three models investigated in this study (Main Text Figs. 7 and 8). However, regressing out the influence of B_1 using Eq. [6] (b) leads to a higher remaining inhomogeneity as shown in Table S3 vs the D2S, KI and KT models for the brains fixed with 10% NBF (Main Text Table 1), and similar performance (no change) for brains fixed with 10% formalin. Results displayed as the mean ± standard deviation across brains.

(a) **10% NBF**

Tissue Type	*Uncorrected*	*B_1 correction*
White Matter	2.80 ± 0.41	2.56 ± 0.34 (0.26)
Grey Matter	6.11 ± 0.77	5.79 ± 0.70 (0.43)

(b) **10% Formalin**

Tissue Type	*Uncorrected*	*B_1 correction*
White Matter	1.46 ± 0.23	1.45 ± 0.21 (0.89)
Grey Matter	2.45 ± 0.32	2.45 ± 0.32 (0.99)

Table S3: **Inhomogeneity over white and grey matter for brains fixed with 10% NBF and 10% formalin – correction with B_1.** Correction with B_1 gave rise to a reduction in inhomogeneity across both grey and white matter for brains fixed with 10% NBF. However, this reduction in inhomogeneity did not reach significance, and was a smaller improvement vs the D2S, KI and KT models (Main Text Table 1). Little change was found in brains fixed with 10% formalin, consistent with the corrections using the D2S, KI and KT models (Main Text Table 1). p-values comparing the change in inhomogeneity for the B_1 correction displayed in brackets.

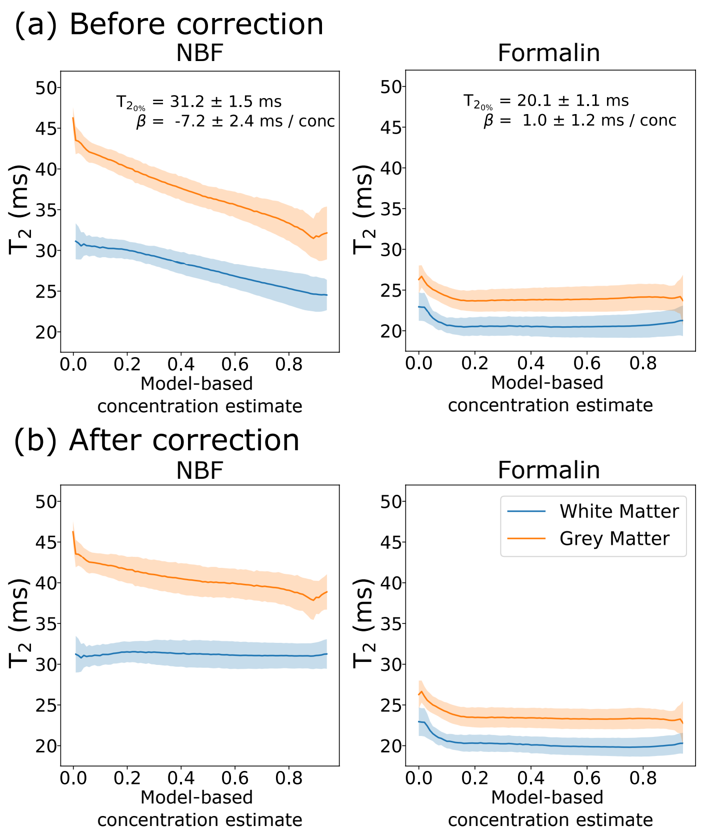

Figure S9: **T_2 vs concentration for the KI model incorporating mean diffusivity maps.** The KT model presented in this work incorporates both diffusion anisotropy and voxel-specific diffusion coefficients to simulate fixative dynamics. Here, we consider how much of the variation in T_2 can be explained by the voxel-specific diffusion coefficients alone. Simulations using the KI model incorporating voxel-wise mean diffusivity maps yields very similar T2-concentration distributions to the KT model (Main Text Figs. 7 and 8). Regressing out the influence of the KI model incorporating these mean diffusivity maps using Eq. [6] (b) leads to a marginally higher remaining inhomogeneity (Table S4) compared to the KT model for brains fixed with 10% NBF (Main Text Table 1), and similar performance (no change) for brains fixed with 10% formalin. This indicates that voxel-wise variations in diffusivity are the predominant driver of fixative dynamics in the KT model, with diffusion anisotropy less crucial for reducing T_2 inhomogeneity. Results displayed as the mean ± standard deviation across brains.

(a) **10% NBF**

Tissue Type	*Uncorrected*	*KI Mean Diffusivity correction*
White Matter	2.80 ± 0.41	2.17 ± 0.16<br>(0.0025)
Grey Matter	6.11 ± 0.77	5.37 ± 0.50<br>(0.055)

(b) **10% Formalin**

Tissue Type	*Uncorrected*	*KI Mean Diffusivity correction*
White Matter	1.46 ± 0.23	1.44 ± 0.21<br>(0.86)
Grey Matter	2.45 ± 0.32	2.47 ± 0.33<br>(0.91)

Table S4: **Inhomogeneity over white and grey matter for brains fixed with 10% NBF and 10% formalin – correction with the KI model incorporating mean diffusivity maps.** Correction with the KI model incorporating mean diffusivity maps led to inhomogeneity estimates similar to the KT model for brains fixed with both 10% NBF and 10% formalin (Main Text Table 1). p-values comparing the change in inhomogeneity for the model correction displayed in brackets.

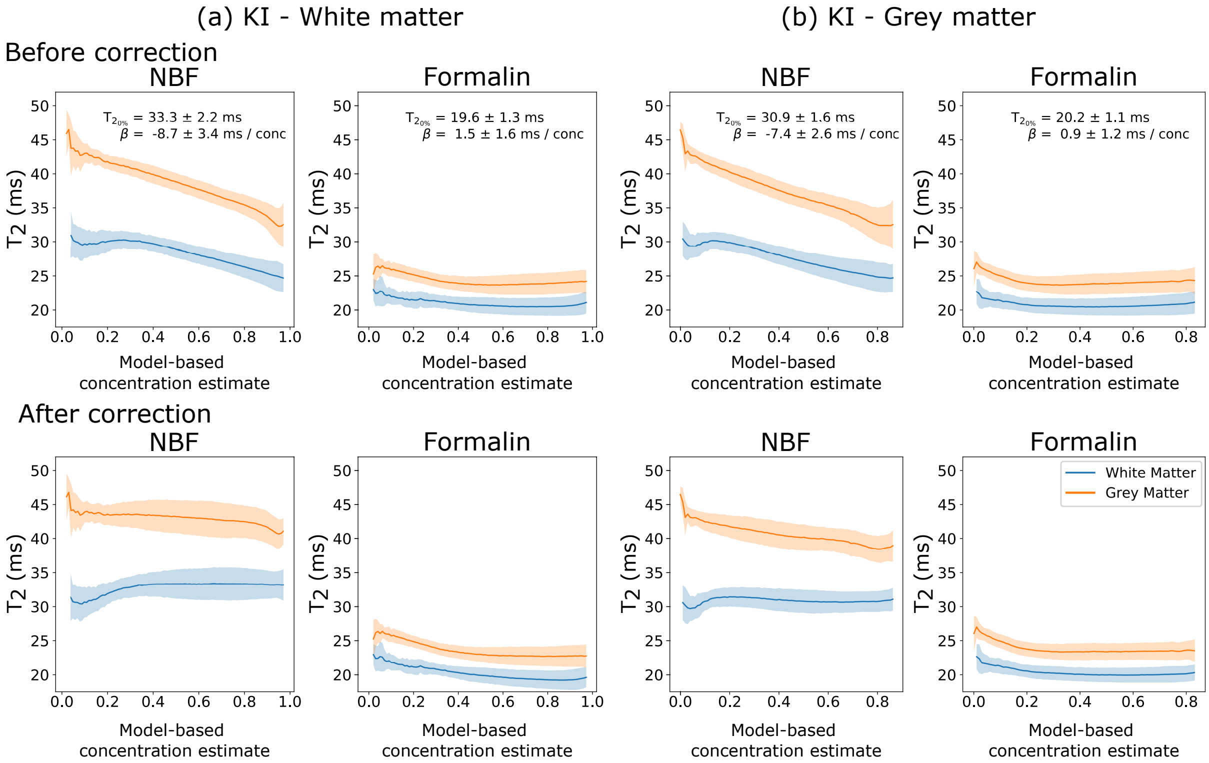

Figure S10: **T_2 vs concentration for the KI model incorporating white and grey matter diffusivity estimates.** The KI model presented in this work simulates fixative dynamics utilising a single, global scalar of diffusivity, set as the average mean diffusivity over the entire brain. Here we repeat the simulations setting the global scalar as the average mean diffusivity over (a) white matter and (b) grey matter. Regressing out the influence of the KI model using Eq. [6] with the average over white matter (a) leads to an improved homogeneity for grey matter for NBF brains, but a considerable reduction in white matter homogeneity verses the original KI model (confirmed in Table S5). Correction with the average over grey matter for NBF brains (b) leads to a slight reduction in homogeneity over grey matter verses the original KI model. Similar performance (no change) was found for brains fixed with 10% formalin. Neither model demonstrated improved performance over the KT model. Results displayed as the mean ± standard deviation across brains.

(a) **10% NBF**

Tissue Type	*Uncorrected*	*KI – white matter correction*	*KI – grey matter correction*
White Matter	2.80 ± 0.41	2.51 ± 0.34 (0.010)	2.25 ± 0.17 (0.0067)
Grey Matter	6.11 ± 0.77	5.39 ± 0.52 (0.064)	5.47 ± 0.53 (0.096)

(b) **10% Formalin**

Tissue Type	*Uncorrected*	*KI - white matter correction*	*KI - white matter correction*
White Matter	1.46 ± 0.23	1.48 ± 0.22 (0.91)	1.44 ± 0.22 (0.87)
Grey Matter	2.45 ± 0.32	2.28 ± 0.20 (0.75)	2.46 ± 0.33 (0.94)

Table S5: **Inhomogeneity over white and grey matter for brains fixed with 10% NBF and 10% formalin – correction with the KI model incorporating white and grey matter diffusivity estimates**. Correction with the KI model incorporating the average mean diffusivity over white matter (middle column) and grey matter (right column). Similar performance was found for brains fixed with 10% formalin verses the KI and KT model (Main Text Table 1). However, the white matter diffusivity leads to an improved homogeneity for grey matter for NBF brains, but a considerable reduction in white matter homogeneity verses the original KI model. Correction with the average over grey matter for NBF brains leads to a slight reduction in homogeneity over grey matter verses the original KI model. Neither model demonstrated improved performance over the KT model. p-values comparing the change in inhomogeneity for the model correction displayed in brackets.

References

1. Hennig J. Echoes—how to generate, recognize, use or avoid them in MR-imaging sequences. Part I: Fundamental and not so fundamental properties of spin echoes. Concepts Magn. Reson. 1991. doi: 10.1002/cmr.1820030302.

2. Hennig J. Echoes—how to generate, recognize, use or avoid them in MR-imaging sequences. Part II: Echoes in imaging sequences. Concepts Magn. Reson. 1991. doi: 10.1002/cmr.1820030402.

3. Weigel M. Extended phase graphs: Dephasing, RF pulses, and echoes - Pure and simple. J. Magn. Reson. Imaging 2015. doi: 10.1002/jmri.24619.
